# Supportive care for men with prostate cancer: why are the trials not working? A systematic review and recommendations for future trials

**DOI:** 10.1002/cam4.446

**Published:** 2015-04-01

**Authors:** Theresa Helen Mazzarello Moore, Anna Jyoti Louise King, Maggie Evans, Debbie Sharp, Raj Persad, Alyson Louise Huntley

**Affiliations:** Academic Unit of Primary Care, School of Social and Community Medicine, University of BristolCanynge Hall, Bristol, BS8 2PA, UK

**Keywords:** Critical appraisal, prostate cancer, randomized controlled trials, supportive care, systematic review

## Abstract

Men with prostate cancer are likely to have a long illness and experience psychological distress for which supportive care may be helpful. This systematic review describes the evidence for effectiveness and cost-effectiveness of supportive care for men with prostate cancer, taking into account treatment pathway and components of interventions. MEDLINE, EMBASE, CINAHL, CENTRAL, and Psychinfo were searched from inception––July 2013 for randomized controlled trials and controlled trials. Two authors independently assessed risk of bias and extracted data. Twenty-six studies were included (2740 participants). Interventions were delivered pre and during (*n* = 12), short-term (*n* = 8), and longer term (18 months) (*n* = 5) after primary treatment. No interventions were delivered beyond this time. Few trials recruited ethnic minorities and none recruited men in same sex relationships. Intervention components included information, education, health professional discussion, homework, peer discussion, buddy support, cognitive behavioral therapy, cognitive restructuring, psychoeducation, Reiki and relaxation. Most interventions were delivered for 5–10 weeks. Risk of bias of trials was assessed as unclear for most domains due to lack of information. The majority of trials measuring quality of life and depression found no effect. Relatively few trials measured anxiety, coping skills and self-efficacy, and the majority found no effect. No cost data were available. Trials of supportive care for men with prostate cancer cover a range of interventions but are limited by population diversity, inconsistent measurement and reporting of outcomes, and inability to assess risk of bias**.** Recommendations on design and conduct of future trials are presented.

## Introduction

Prostate cancer is the second most common cancer worldwide for men, with an estimated 900,000 new cases diagnosed annually 1. A large increase in incidence has been reported in recent years with much of this increase being attributed to increased prostate-specific antigen (PSA) testing 2,3.

Men with prostate cancer are likely to have a long illness pathway with the greater part being supported by family, friends, and general practitioners. The National Cancer Institute defines the goal of supportive care as “to prevent or treat as early as possible the symptoms of a disease, side effects caused by treatment of a disease, and psychological, social, and spiritual problems related to a disease or its treatment” 4.

Qualitative research tells us that supportive care is wanted by patients but that it is felt there is a lack of appropriate support services 5. A recent survey covering seven European countries and involving over 1000 men suggests that 81% of the respondents had some unmet supportive care needs including psychological, sexual and health system, and information needs 6.

There are four previous relevant reviews 7–10. One was a narrative 2010 review focusing on the nature and content of the 17 included studies, in order to advance understanding of self-management of men with prostate cancer, and did not focus on the outcome data 7. Two systematic reviews looked at psychosocial interventions for men with prostate cancer 8,9. The Chambers 2011 review was narrative and briefly described the outcome data of 21 studies and concluded that the research was limited on effective ways to improve adjustment for men with prostate cancer 8. The Chien 2012 review sought to combine data from 14 studies in meta-analyses and concluded that psychosocial approaches reduced anxiety and depression 9. A recent 2013 Cochrane review and meta-analysis by Parahoo included 19 psychosocial interventions and concluded that there was evidence that psychosocial interventions improve quality of life (QoL) 10.

This review aimed to include supportive care interventions using broader inclusion criteria than these four previous reviews. Our review comprising 26 trials included psycho-social and self-management approaches but also extended to include trials of relaxation, music therapy, basic information provision, and peer support interventions. This review was designed to take into account the patient pathway and to identify the individual components of the interventions with the purpose of determining which therapeutic components were contributing to the success of an intervention. To our knowledge, this approach had not been conducted within a rigorous systematic review of supportive care for men with prostate cancer.

## Methods

### Eligibility criteria

The review included both randomized controlled or controlled trials (RCTs, CTs) that involved men with a diagnosis of prostate cancer; undergoing or having under gone any type of standard treatment, including active monitoring. The review did not include trials of men at risk from prostate cancer, men with advanced cancer or those who were in the last days of life.

Any intervention was included in the review that could broadly be defined as supportive, but trials of pharmaceutical interventions including herbal medicine and nutritional supplements, and trials of treatment decision aids were excluded.

Trials in all languages were included as long as the abstract was in English in order to assess eligibility. If a trial involved a mixed population of cancer patients in which the data for prostate cancer patients could not be separated it was excluded. The outcomes of interest were QoL and wellbeing, psychological health, health behaviors, physical health, and cost-effectiveness data. Outcomes directly relating to family or partners were not collected.

### Information sources and searches

The databases MEDLINE, EMBASE, CINAHL, CENTRAL, and Psychinfo were searched from their inception to July 2013 using custom-designed search strategies which combined terms of prostate cancer, supportive care interventions, and study type (Appendix S1). The reference lists of all the included studies were screened for additional relevant papers and key authors were contacted regarding any unpublished studies.

### Study selection

The references retrieved from the searches were downloaded into Endnote X6 and were managed using a customized Access 2010 database. All titles and abstracts from the searches were screened using the eligibility criteria and any studies selected were obtained in full and assessed in detail by two reviewers in duplicate and independently (TM,AH). Reasons for exclusion of all full text trials were recorded in the Access database.

### Data extraction and risk of bias

Data were extracted on study details, participant characteristics, outcome measures, and results. The intervention components were classified as information-based (*information, education, health professional discussion, homework*), peer support (*peer discussion, formal buddy system*) or as specific therapeutic approaches (*cognitive behavioral therapy [CBT], cognitive restructuring [CR], psycho-education, Reiki, relaxation*) (Appendix S2).

One researcher independently extracted data and assessed risk of bias using the Cochrane Collaboration’s risk of bias tool and a second checked the accuracy 11.

Each study was assessed in the following domains: random sequence generation, allocation concealment, blinding of participants and personnel, blinding of outcome assessment, incomplete outcome data, selective reporting, and other possible sources of bias. The terms “high risk,” “low risk,” and “unclear risk” were used to rate the level of bias. The decisions of a researcher had to be supported by evidence or lack of evidence from the published material. In all the screening, data extraction and risk of bias assessment, disagreements were resolved by consensus and where necessary recourse to a third reviewer. The writing of the review followed PRISMA guidelines (http://Prisma-statement.org).

### Synthesis of results

The trials were classified into four groups (Appendix S3)

1. Pre and during primary treatment,
2. Short term after primary treatment (≤6 months),
3. Long term after primary treatment (>6 months),
4. All stages.

Effect sizes were calculated and presented in forest plots as mean differences or standardized mean differences when data were available, and when effect sizes for a forest plot could not be calculated the author’s data were reported.

## Results

### Studies identified

**Figure 1:**
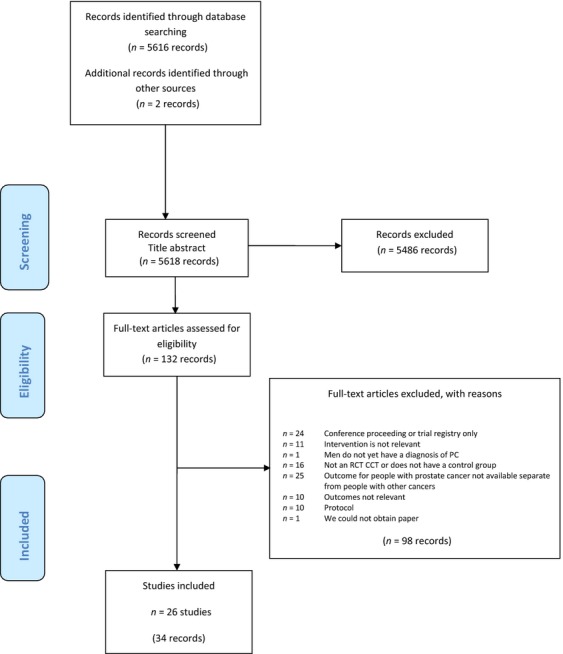
PRISMA flow chart of review.

The review identified 34 papers that described 25 RCTs and one CT which included a total of 2740 participants 12–45 (Fig.1). There were 12 trials conducted pre or during treatment (1173 participants) 12,14,16,18–21,23,26–29, eight conducted in the short term following treatment (824 participants) 30,31,33–35,37–39, five conducted longer term following primary treatment (743 participants) 40–44, and one trial which included patients at all stages of pathway (263 participants) 45 (Tables S1 and S2).

### Risk of bias

Overall, information from the included trials needed to assess risk of bias was poor and therefore the trials were graded as unclear for most domains. In supportive care trials, it is not possible to blind participants to their allocation therefore all trials were graded at high risk for this domain. One trial was a CT and was rated at high risk for all domains 20. There were 13 trials which reported how incomplete data were managed and so they were rated as low risk, six were rated at unclear risk, and seven at high risk for this domain Table  S2.

### Provenance and size of trials

Twenty trials were conducted in the United States, two in Canada, and one each in Northern Ireland, Sweden and Hong Kong. The U.S. and Canadian trials were generally funded by government or national research bodies, the remaining trials were funded by a mixture of public and private funding, and five of the trials did not declare a funding source.

In terms of number of trial participants, eight trials had less than 50, six trials had 50–100, six trials had 101–200, and the six remaining trials had greater than 200 participants. Seven trials were described as pilot studies 12,27,28,30,34,37,40, of which two were not described as “pilot” until the discussion or the main study paper 30,34. Power calculations were reported in four of the 19 “full scale” trials 20,21,23,45 and in one of these the participants included prostate cancer and breast cancer patients in the calculation 20.

### Control groups

There were 16 trials that described the control group as usual, standard, routine care or as a “wait list” 12,14,20,21,23,28–31,33–35,38–40,45. One trial gave no detail at all 26. In nine of the trials, the control participants received services or support in addition to usual care that were not classified as an intervention 16,18,19,27,37,41–44.

### Baseline characteristics of participants

The trial participants were on average in their 60s, married or partnered except for one trial in which 59% of men were not partnered 27. There were two trials that did not report marital status 40,42. There were seven trials that included men from ethnic minority groups 21,27,40–45. In all trials, the majority of participants had received or was receiving an interventional treatment except in one trial which recruited patients with an average age of 75 years who were being actively monitored 30.

### Delivery of interventions

There were 14 interventions that were delivered to the individual 12,16,18–21,23,26–30,38,39, Eight interventions involved couples or family members. 22,28,33–35,40–45 and eight interventions were delivered to groups 14,31,34,35,41–44. Ten of the interventions were individualized 21,23,26–30,33,37–39. The majority of interventions were delivered or facilitated by health professionals: nurse (6), psychologist (5), mixed-health professionals (4), “medically trained” (1), researcher (5), peers (2), volunteers/minimal training (2), and no provider (1). The majority of trials (22/26) were delivered in person but some also involved phone calls or emails. There were four interventions that were delivered exclusively by telephone 21,27,30,40. Three of the included trials described interventions with homework 12,14,41.

Most of the interventions were short in duration and intensity with a general length of between 5 and 10 weeks, and weekly meetings of 1–2 h. The range of duration was 2–48 weeks, with the longer term interventions generally becoming less intense with monthly contact or contact by phone.

### Components of interventions

#### Pre or during treatment

Nine of the 12 trials investigated intervention(s) comprising at least one informational component 12,14,16,18–20,23,26,28. Four of these nine trials comprised information-based components only 16,18–20,26,28. Six of the 12 trials investigated intervention(s) comprising at least one specific therapeutic approach 12,14,21,23,27,29. Only one intervention had a component of peer support 23.

#### Short term following treatment

The eight trials investigated intervention(s) comprising a range of components. Four of these eight trials mainly comprised psychoeducation, education and discussion with health professionals 30,31,33,37. The remaining four trials investigated interventions of peer support facilitated by health professionals 34,35,38,39.

#### Longer term following primary treatment

Four of these five trials described the same stress management intervention which comprised information-based, specific approach (CBT), and peer support components 41–44. The remaining trial investigated a coping skill intervention which comprised informational and specific approach components 40.

#### Patients at all stages of pathway

One trial described a psycho-education intervention for couples first developed for breast cancer patients and spouses, and modified for prostate cancer 45 (individual intervention components detailed in Table S1).

### Outcomes

The most frequent outcomes measured in these trials were QoL, depressive symptoms, anxiety, and to a lesser extent coping and self-efficacy. Only eight of the 26 trials distinguished between primary and secondary outcomes 12,14,23,28,33,35,37,45 (all individual data are in Table S1).

Of these eight, only four studies showed improvement in one of their primary outcomes 12,23,33,35. None of the 26 studies explicitly stated a primary endpoint.

Researchers measured and reported a wide range of other patient outcomes related to these main outcomes such as uncertainty management, wellbeing, life orientation, and pain but none showed any improvement.

#### Quality of life

Overall, 22 of the 26 trials measured QoL with 15 using general or general cancer QoL scales and 10 using prostate cancer-specific scales. Five of the 22 trials explicitly stated that QoL was a primary outcome. Seven of the 22 trials reported an improvement in QoL in the intervention group compared with the control group 23,30,33–35,41,44. Two of those 10 trials explicitly stated a prostate cancer-specific QoL scale as a primary outcome measure 33,35. Examination of effects using a forest plot of standardized mean differences where data were available indicated that there was no consistent direction of effect with QoL measures (Fig. S2A–G).

##### Pre or during treatment

Six of the 12 pre and during treatment trials measured QoL and used either the Short Form 36-item (SF-36) or the Functional Assessment of Cancer Therapy–general 27-item (FACT-G-27) scales 12,14,20,23,26,27. One study by Parker demonstrated that men given a presurgical stress management intervention had improved QoL as measured by the SF-36-physical component score compared to the control group at 12 months (p=0.0009) 23.

##### Short term following treatment

Four of the eight short-term trials measured general QoL, using SF-36, and a further trial used Cantril’s ladder (authors own measure using a visual analog scale) 30,33–35,39. One trial measured QoL using the cancer-specific score of the European Organisation for Research and Treatment of Cancer Quality of life C30 31.

Two of the trials reported improvements in general QoL with a supportive care intervention compared to control groups. Lepore 35 reported that a psycho-educational support group had improved mental health scores using the SF-36 scale compared to controls 2 weeks postintervention (*P* = 0.05) 34. Bailey used Cantril’s ladder and reported that QoL was greater for men receiving an uncertainty intervention compared to controls at 10 weeks follow up p = 0.006 30.

Four of the eight short-term trials reported prostate cancer-specific QoL and used either the UCLA Prostate Cancer Index 20-items (UCLA-PC-20) or the Prostate Cancer Quality of Life Instrument 52-items (PCQoL-52) 33,35,38,39. Two of these four trials reported an improvement. In Lepore et al. 36 reported that group education with discussion resulted in participants being less bothered by sexual problems than those in the control group. Post hoc analyses indicated that sexual bother within the primary outcome measure of the UCLA-PC-20 index was significantly worse in the control group than in the education-plus-discussion group (*P* < 0.01) 35. In the Giesler trial, the nurse-led computer program-based intervention resulted in a reduction in sexual limitation using the primary outcome measure of the PCQoL-52 compared to the control group at both four and 7 months (*P* = 0.05; *P* = 0.02, respectively), and showed significant reduction in cancer worry at 7 months (*P* = 0.03) 33.

##### Longer term following treatment

Three of the five longer term trials investigating the same CBT-based intervention measured QoL using the FACT-G-27 score 42–44. All three showed some positive effect on QoL with the intervention pre and postintervention but only the Traeger study gave data in comparison with controls (*P* < 0.01) 42–44. Campbell measured QoL using SF-36 and reported no differences between telephone-based coping skills training and control groups 4.

Both Traeger and Molton also looked at sexual functioning measured by the Expanded Prostate Cancer Index Composite 26 items and UCLA-PC-20 scales, respectively 41,44. Traeger showed no benefit but Molton reported a significant improvement in sexual functioning with the intervention in comparison with the control group (37.4% vs. 11.5%) 41.

##### All stages

Northouse measured general (SF-12), cancer-specific (FACT-G), and prostate cancer-specific (FACT-P) QoL and reported that there were no improvements for the men following a supportive education program compared to the control group 45.

#### Depressive symptoms, mood, and anxiety

Overall, 14 of the 26 trials used depressive symptoms or mood as an outcome and three trials measured anxiety with a separate measure. In the 14 trials, three showed an improvement in the intervention group compared with the control group 23,38,39. One of these three trials explicitly stated mood as a primary outcome measure 23. There were no trials that showed a positive intervention effect on anxiety. Examination of effects using a forest plot of standardized mean differences where data were available indicated that although many of the studies found no effect on depressive symptoms, mood or anxiety, and the confidence intervals were generally wide they are tending toward a positive effect (Fig. S3A–C).

##### Pre and during treatment

Depressive symptoms were measured by the Centre for Epidemiologic Studies Depression scale (CES-D-20) or Profile of Mood States (POMS, various numbers of items used) in seven of the 12 included trials 12,14,16,18–20,23. None of the studies show any significant effect on depressive symptoms with the exception of the Parker trial 23. In this trial of a presurgical stress management intervention mood (a primary outcome) measured by POMS-18 item improved in the intervention group compared to the attention control group at 1 week before surgery p = 0.006. However, by the morning of surgery there were no differences between the groups 23. Three studies investigating relaxation, lifestyle, and education interventions, respectively, measured anxiety using the State Trait Anxiety Index-20 items and reported no improvement in anxiety compared to control groups 12,14,28.

##### Short term following treatment

Seven of the eight included trials measured depressive symptoms using either the Hospital Anxiety and Depression Scale 14-item (HADS-14), Geriatric Depression scale 15-item (GDS-15), CES-D-20, or POMS-various items 30,31,34,35,37–39. Two of the trials showed a statistically significant improvement albeit short-term with a supportive care intervention in comparison to a control group. These two trials were a pilot and a main trial of a trained buddy support intervention 38,39. The pilot trial showed significant improvement in depressive symptoms at 4 weeks with the intervention compared to the control group, using the GDS-15 p = 0.014 38. In the full-scale trial, the intervention group had significantly lower depressive symptoms (GDS-15) at 8 weeks compared with the control group (*P* = 0.03).

Two studies found relaxation therapy had no effect on anxiety (HADS-14) compared to controls 31,37. There were no data from longer term or all stage trials.

#### Coping skills and self-efficacy

Four trials each used coping and self-efficacy as an outcome measure 16,18,26,34,38–40,45. In none of these studies were the outcomes explicitly stated as primary or secondary. There were insufficient data to produce forest plots for the outcomes of coping and self-efficacy.

##### Pre and during treatment

Two studies found that “concrete information” provided to participants, prior to their treatment, improved coping skills as measured by the Sickness Impact Profile-136 item (SIP-136) compared to a control group over 3 months (*P* < 0.02, *P* < 0.05, respectively) 16,18. One of these studies had an additional intervention group of providing participants with coping *and* self-care information which did not improve coping skills compared to the control group 18. In the Templeton trial, the intervention group received an evidence-based education package. Coping was measured using the 40-item Jalowiec coping scale, however the data provided by the authors was not in a usable format.

##### Short term after treatment

One study showed that psycho-educational support increased self-efficacy measured by the investigators own scale compared with the control group 2 weeks postintervention (*P* < 0.05) 34. Self- efficacy measured by the Stanford Inventory for Cancer Patient Adjustment scale 38-items was not improved by a trained peer buddy intervention compared to controls 38,39.

##### Longer term after treatment

There were limited data in the longer term, with one trial showing that self-efficacy as measured by the Self Efficacy for Symptom Control Scale–13 items was not improved by providing supportive educative home visits compared to controls 40.

##### All stages

A supportive educational home visit delivered to participant spouse dyads did not improve coping skills as measured by the Brief Coping Orientations to Problems Experienced scale 28-items compared to standard clinic care 45.

#### Costs and cost analysis outcomes

None of the included trials described any costs or cost analysis.

## Discussion

This systematic review included 26 papers describing supportive care interventions for men with prostate cancer. All of the trials rated poorly overall in terms of risk of bias or provided too little information for a judgment of bias to be made. Whilst we recognize that many of the trials found positive effects of their intervention on specific outcomes and various follow-up, overall the picture is more temperate.

The most frequent outcomes measured in these trials were QoL, depressive symptoms, anxiety, coping, and self-efficacy. Only seven of the 22 trials measuring QoL reported an improvement in the intervention group compared with the usual care group. Fourteen of the 26 trials used depressive symptoms or mood as an outcome, and three showed an improvement in the intervention group compared with the control group. Three trials measured anxiety as an individual measure and no trials showed a positive intervention effect. There were insufficient data on coping and self-efficacy.

The interventions were often complex comprising several components, for example, Campbell 2007 described an intervention of education, CR and relaxation therapy delivered to couples. Our original aim to determine which of the components of these interventions were contributing to a positive treatment effect was not possible due to the limited evidence. However, it was possible to distinguish a changing profile of intervention components across the patient treatment pathway covered in the included trials. Information and education featured prominently in the trials of interventions around the time of treatment. In the short and longer term studies, peer support and psychologically based intervention components dominated. These approaches tally with the supportive care needs described by men with prostate cancer in qualitative studies 46,47.

In light of a lack of robust evidence for supportive care interventions for men with prostate cancer, it is important to examine why this was the case. This examination took into account that the trials considered the patient pathway in their recruitment and appeared to investigate appropriate interventions and measure a range of appropriate outcomes. Thus, we have compiled a list of recommendations for future trials based on our critique of the included studies (Box 1).

**Box 1 Recommendations for future trials of supportive care interventions for men with prostate cancer**

Overall

**High quality design and conduct of trials**

Local guidance should be followed

http://www.mrc.ac.uk/documents/pdf/good-clinical-practice-in-clinical-trials/ (UK)

http://www.fda.gov/ScienceResearch/SpecialTopics/RunningClinicalTrials/GuidancesInformationSheetsandNotices/ucm219488.htm (USA)

Power calculations made to ensure trials sufficiently powered relevant to outcomes of interest

http://www-users.york.ac.uk/~mb55/msc/trials/sampsz.htm

Include a nested qualitative investigation into trials to examine participants and partners experiences of the intervention.

Barbour, R. S. 1999. The case for combining qualitative and quantitative approaches in health services research. *J. Health Serv. Res. Policy*. 4:39–43.

**High quality of reporting of trials**

Authors should report the studies following CONSORT guidelines

http://www.consort-statement.org/

**Collection of costs and cost/benefit analysis**

Whilst determining the effectiveness of an intervention is important, there is little chance of an intervention being implemented by commissioners of care without relevant accurate costs and cost analysis.

Specifically

**Patients**

To conduct trials to represent all men in terms of ethnicity, sexuality and partnership status. The majority of trials to date focus on white, partnered men. The supportive care needs of men are likely to be influenced by men socio-demographic profile 51–53.

To involve or consider the support of a partner close family or friend in trials. Qualitative research tells us that this type of support is integral to any external support that men with prostate cancer receive 50.

**Interventions**

To conduct trials that examine all stages of the treatment pathway especially the longer term and patients being active monitored 6.

**Control groups**

Usual care or control groups need to be well described to be able to determine what the intervention adds.

**Outcomes**

Standardization of outcomes to allow comparisons across studies and combination of data in systematic reviews 52

Appropriate, validated outcomes to capture relevant issues for men with prostate cancer. For example, prostate cancer-specific quality of life measures.

Measures which can capture accurately capture the extent of depression

### Limitations of the included studies

#### Population

Overall there were few trials recruiting ethnic minorities and there were no studies on younger men with prostate cancer, no studies explicitly investigating supportive care needs of men in same sex relationships or men without partners. Men with different ethnic and or socio-demographic backgrounds are likely to have different supportive care needs 48–50. Appropriate tailoring of interventions is not possible without evidence from studies including or focusing on these groups of men.

#### Intervention

All the trials included in this review were limited in terms of timing and duration. Men with prostate cancer were generally only recruited in the period preceding and following primary treatment. There were no trials recruiting men beyond 18 months postprimary treatment with the exception of the Bailey trial 30. The average length of an intervention was between 5 and 10 weeks. The few trials of longer duration tended to be low intensity and tapered. The most likely explanation for this is that long-term trials are expensive and are likely to be subject to attrition which will diminish their impact. However, qualitative data tells us that men’s supportive care needs continue throughout their lives and therefore we need more evidence on the longer term care of men to determine which approaches are likely to be most effective and cost-effective 46.

There was a range of delivery of the interventions across individuals, groups of men, and couples. In a recent review, it was reported that there is a lack of evidence to support the idea that delivery to a group of men as opposed to an individual was beneficial 10. Nevertheless, qualitative studies show that an informal support network, including partners and peers is important to men 46,47. It would be useful for future studies to investigate the format of delivery to provide a more robust evidence base for this aspect of supportive care.

#### Control groups

In many of the studies, the control group of usual care was not defined. In a minority of cases, the control groups received some of the intervention components. Without the detailed knowledge of baseline usual care or what services patients have access to, it is difficult to assess what an intervention will provide in addition. It is of note that most of these trials were conducted in the United States. Other countries are likely to have different levels of standard care. Thus, all new studies should provide an adequate description of the usual or standard care of the patients to give an accurate baseline to additional supportive care. It is also important to consider that standards of care are intrinsically linked to socioeconomic status, and are likely to differ between affluent and more deprived areas, and urban and rural environments.

#### Outcomes

Data presentation was poor in many papers. Raw data was often not reported nor available from the authors. In many cases, the editors of the journals in which they are published have not required trials to be reported following CONSORT guidelines 51. Although not all journal editors enforce this, it has made the assessment of risk of bias difficult and largely the studies are reported as unclear or at high risk of bias 52.

Conducting systematic reviews and producing evidence-based recommendations relies on good reporting from clinical trials. Without the essential information and data being reported, reviewers are obliged to concluded that the conduct of the trial is of unknown quality when in fact it may be either at high or at low risk of bias 51–53.

A qualitative synthesis of studies of men with prostate cancer describing their experiences of supportive care and unmet needs has been conducted alongside this intervention review 47. In light of these data, it is possible to suggest that the majority of studies in this review covered the most relevant outcomes to men with prostate cancer. The studies in the qualitative review describe men’s experiences of dealing with reduced QoL and life-changing side effects, depressive feelings, and anxiety of waiting for treatment and PSA testing 47. Although many of the trials measured QoL, some trials used only a general or cancer QoL measures, with fewer using a prostate cancer-specific scale. All the QoL measures used in the trials are in common use and have been validated. Whilst some QoL issues are common to all (cancer) patients, the prostate cancer-specific measures capture the important impact of urinary and sexual dysfunction which many men with prostate cancer experience 47.

Overall, the trials that used outcome measures of depression aimed to look at depressive and anxiety symptoms as opposed to clinical depression, with only two trials showing a small percentage of clinically depressed patients^12^,^33^ and four studies using clinical depression or the use of antidepressants as an exclusion criteria 12,20,23,35. A retrospective cohort study of over 50,000 patients with prostate cancer reported that 8.54% of these men had depression diagnosed using the *ICD-Ninth Revision Clinical Modification* 54. The authors also report that depression was associated with higher odds of secondary care use (and costs) and greater risk of death. Thus, the trials included in the review most likely underrepresent the prevalence of men with prostate cancer and depression. Although the measures in the included trials are well used and validated, for example, CES-D-20, POMS; they are not adequate to diagnose major depression. Furthermore, the short duration (6 months or less) of the majority of the trials would not capture any new, recurrent or prolonged depression associated with any ineffective primary or subsequent treatments.

Previous research by Sharpley et al. proposes five depressive subtypes within patients with prostate cancer: melancholic, depressed mood, anhedonic, somatic and cognitive 55. These subtypes describe a wide range of beliefs and attitudes within the diagnosis of depression and highlights the need for a complementary wide range of approaches to treat depression in men with prostate cancer. None of the trials included in this review tackled depression or low mood in this targeted way.

Coping and self-efficacy are applied outcomes of the above but unfortunately data were limited and mostly negative. The less prevalent outcomes were measured by a mixture of validated, unknown, and author’s own measures.

The decision was made by the team not to combine the data in meta-analysis due to heterogeneity. Whilst the sample of men is homogenous, the interventions are heterogeneous in terms of content and when, how, why they were delivered, for example, Reiki for acute stress, peer support in cafes. Although there were similar outcomes and outcome measures used from which data may have been combined, few data were available in a usable form or in a similar follow up time. This lack of standardization meant that combined data was unlikely to be clinically meaningful. There is currently research based in Newcastle, UK., as part of the COMET initiative to develop a set of core outcome measures (COMs) for use in advanced prostate cancer trials. The development of such a set of standardized outcomes will progress the evidence base more rapidly 56.

#### Design

Whilst there were some larger studies, many were small and most likely not powered sufficiently to determine differences. Whilst some studies were described as pilot studies, in those that were not, the majority did not report a power calculation or the calculation was inappropriate.

#### Costs

In order to gain funding for or commissioning of supportive care for men with prostate cancer, cost-benefit must be determined. There were no costs or cost-effectiveness data in any of the trials included in this review.

## Conclusions

Published trials on supportive care for men with prostate cancer appear to provide appropriate interventions and measure appropriate outcomes, but provide insufficient evidence to improve men’s experiences. Trials do not always use appropriate outcome measures, and have focused on limited patient groups and stages of the patient pathway. The majority of trials measuring QoL and depression found no effect. Relatively few trials measured anxiety, coping skills and self-efficacy, and the majority found no effect. No cost data were available. Many of the studies were small and were likely to be underpowered to detect a difference. Detailed assessment of these trials has resulted in a list of recommendations for future trials.

## Supporting Information

**Appendix S1.** MEDLINE Search on OVID 1950 to Present 2013-05-02

**Appendix S2.** Definition of Intervention Components

**Appendix S3.** Classification of Studies by Patient Treatment Pathway

**Figure S2.** Forest plots summarizing available data from trials with outcomes of QoL. (A) Various QoL up to 12 months; (B) SF-36 MCS 2 weeks; (C) SF-36 PCS 2 weeks; (D) SF36 MCS 6 weeks to 6 months; (E) SF36 PCS 6 weeks to 6 months; (F) SF36 MCS 6 to 12 months; (G) SF36 PCS 6 to 12 months.

**Figure S3.** Forest plots summarizing available data from trials with outcomes of depressive symptoms, mood and anxiety. (A) Depressive symptoms; (B) mood; (C) anxiety.

**Table S1.** (A–D) Supportive care interventions for men with prostate cancer for (A) pre and during primary treatment, (B) short term after primary treatment, (C) longer term postprimary treatment, and (D) studies involving men at all stages of prostate cancer & treatment.

**Table S2.** (A) Risk of bias tables for all studies.
