# Supplementary material for: Supportive care for men with prostate cancer: why are the trials not working? A systematic review and recommendations for future trials
Source: Cancer Med. 2015 Apr 1;4(8):1240–51. doi: 10.1002/cam4.446 (PMC4559035; doi:10.1002/cam4.446)
Supplement: Supplementary file 1 [file cam40004-1240-sd1.docx]

**Appendix 1: MEDLINE search on OVID 1950 to present 2013-05-02**

--------------------------------------------------------------------------------

1 exp prostatic neoplasms/ (85724)

2 prostatic intraepithelial neoplasia/ (1146)

3 (prostat* adj4 cancer*).tw. (68831)

4 (prostat* adj4 neoplas*).tw. (2952)

5 (prostat* adj4 carcinoma*).tw. (15356)

6 (prostat* adj4 tumo?r*).tw. (9931)

7 1 or 2 or 3 or 4 or 5 or 6 (99504)

8 Adaptation, Psychological/ or psychosocial*.mp. (116994)

9 Rehabilitation/ (16182)

10 (supportive adj care).tw. (7328)

11 (supportive adj measures).tw. (1278)

12 supportive care program*.tw. (27)

13 (supportive adj care adj3 management).tw. (143)

14 (support adj3 group*).tw. (7557)

15 *Social support/ (16538)

16 or/8-15 (158988)

17 Self care/ (21763)

18 *Self-Help Groups/ (4260)

19 (self adj direct*).tw. (2774)

20 (self adj efficacy).tw. (10317)

21 (self adj manag*).tw. (6855)

22 (coping adj skills).tw. (1570)

23 (self adj3 care).mp. (27675)

24 or/17-23 (47562)

25 Psychotherapy/ (39664)

26 exp psychotherapy/ (143356)

27 exp Counseling/ (31315)

28 (talk* adj3 (therap* or treat*)).tw. (264)

29 psychotherap*.tw. (28719)

30 (psycholog$5 adj intervent$5).tw. (2042)

31 psychologic*.tw. (121562)

32 ((behavio* adj3 therap*) or (cognitiv* adj3 therap*) or cbt).tw. (16278)

33 (behavio?r*4 adj4 (modify or modifica*4 or therap*2 or change)).tw. (29866)

34 exp Cognitive Therapy/ (14056)

35 marital therapy.mp. or exp Marital Therapy/ (1443)

36 couple* therap*.mp. or exp Couples Therapy/ (593)

37 Sex counseling/ or sex therap*.mp. or sex counse*ing.tw. (1266)

38 or/25-37 (304956)

39 exp Mind-Body Therapies/ (39328)

40 Relaxation/ or Relaxation Therapy/ or Meditation/ or Mindful?ness.mp. (9049)

41 (relaxation adj5 (treat* or therap* or technique*)).tw. (3808)

42 (mindful* or (third adj wave)).tw. (2180)

43 guided imagery.tw. (430)

44 (supportive adj3 therap*).tw. (3952)

45 Spiritual Therapies/ (417)

46 Yoga/px, th [Psychology, Therapy] (168)

47 Tai Ji/ or tai chi.mp. or qigong.mp. or chi kung.mp. or ai chi.mp. (946)

48 Acupuncture Therapy/ or Acupuncture/ or Aromatherapy/ or Complementary Therapies/ or Homeopathy/ or Massage/ (32098)

49 or/38-48 (353595)

50 Patient education as Topic/ (66878)

51 Health education/ (50537)

52 Information services/ (14802)

53 or/50-52 (128524)

54 *"Online Systems"/ (2993)

55 online.tw. (26379)

56 *Electronic Mail/ut [Utilization] (97)

57 web-based.tw. (10976)

58 (online adj3 support*).tw. (474)

59 or/54-58 (37775)

60 Herbal Medicine/ (1284)

61 Phytotherapy/ (26813)

62 Plants, Medicinal/ (50267)

63 Plant Preparations/ (5956)

64 Plant extracts/ (62676)

65 Dietary supplements/ (27315)

66 *Cimicifuga/ae, de (38)

67 (Black adj cohosh).tw. (314)

68 *Lycopersicon esculentum/ae, tu (22)

69 Lycopene.tw. (2789)

70 *Vitamin D/ae, tu, th (2159)

71 (vit* adj D).tw. (32389)

72 *Drugs, Chinese Herbal/tu (6161)

73 (pc-spes or pc-hope or pc-care).tw. (107)

74 or/60-73 (179124)

75 exp Exercise/ or Physical Fitness/ (115311)

76 Exercise Therapy/ or Exercise Tolerance/ or Exercise Test/ or exp Sports/ or Motion Therapy, Continuous Passive/ or Muscle Stretching Exercises/ or Plyometric Exercise/ or Resistance Training/ or Pliability/ (171654)

77 exercising.mp. (6777)

78 physical condition*.mp. (12440)

79 stamina.mp. (342)

80 pilates.mp. (91)

81 ((core or trunk) adj (strength or stability)).mp. (366)

82 resistance training.mp. (4396)

83 pelvic floor.mp. (5872)

84 kegal.mp. (0)

85 or/8-84 (1036431)

86 randomized controlled trial.pt. (347234)

87 controlled clinical trial.pt. (85791)

88 randomi?ed.tw. (318012)

89 placebo.tw. (141318)

90 clinical trials as topic.sh. (164147)

91 randomly.tw. (179200)

92 trial.ti. (107077)

93 or/86-92 (822556)

94 exp animals/ not humans/ (3806377)

95 93 not 94 (757640)

96 7 and 85 and 95 (786)
