# Supplementary material for: Supportive care for men with prostate cancer: why are the trials not working? A systematic review and recommendations for future trials
Source: Cancer Med. 2015 Apr 1;4(8):1240–51. doi: 10.1002/cam4.446 (PMC4559035; doi:10.1002/cam4.446)
Supplement: Supplementary file 2 [file cam40004-1240-sd2.docx]

**Appendix 2: Definition of intervention components**

| **Classification** | **Definition** |
| --- | --- |
| Interaction with health professionals |  |
| Information | Health professionals delivering information in any format with no or little explanation or discussion. |
| Education | Health professionals delivering information in any format with explanation and or discussion. |
| Discussion | Health professionals discussing any aspect of prostate cancer, its treatment and the consequences of both. |
|  |  |
| Interaction with peers |  |
| Trained buddy | Support from an experienced patient with prostate cancer (further down the cancer/treatment pathway) to another patient who is newly diagnosed/only just having those experiences. The experienced patient (buddy) is identified by a health professional and is given training by health professionals |
| Discussion | Discussion between patients with prostate cancer usually facilitated by health professionals or researchers as part of an intervention |
| Other |  |
| Reiki | Reiki is a type of complementary therapy used to improve the body’s ability to heal itself. |
| Relaxation | Therapy that teaches subjects to evoke the relaxation response. |
| Music | Music used as therapy in order to have the effect of reducing activity in the autonomic nervous system and producing a relaxation response as well as a sense of well-being. |
| Cognitive behavioural therapy (CBT) | Psychotherapeutic approach that addresses unhelpful thoughts, emotions, behaviors and cognitive processes and contents through a number of goal-oriented, explicit systematic procedures. |
| Cognitive restructuring | Psychotherapeutic approach to improve the ability to address concerns about illness from a positive point of view. |
| Psycho-education-men | Education delivered to individual men in order to reduce distress by providing participants with effective coping and stress management techniques, as well as practical information |
| Psycho-education- couples | Education delivered to couples in order to reduce distress by providing participants with effective coping and stress management techniques, as well as practical information |
