# Supplementary material for: Supportive care for men with prostate cancer: why are the trials not working? A systematic review and recommendations for future trials
Source: Cancer Med. 2015 Apr 1;4(8):1240–51. doi: 10.1002/cam4.446 (PMC4559035; doi:10.1002/cam4.446)
Supplement: Supplementary file 3 [file cam40004-1240-sd3.docx]

**Appendix 3: Classification of studies by patient treatment pathway.**

| **Classification** | **Definition** |
| --- | --- |
| **On** **diagnosis** | of prostate cancer at any stage of the disease, |
| **Pre-treatment** | Post diagnosis of prostate cancer but before a primary treatment. |
| **During treatment** | Receiving any conventional (primary) treatment for prostate cancer. |
| **Active monitoring only**  (watchful waiting) | Close monitoring of prostate cancer for any changes. No medical treatment provided but periodic tests done to check for signs the cancer is advancing. |
| **Short term after treatment** | Post treatment and up to and including 6 months post treatment. |
| **Long term** **after treatment** | 6 months and beyond after treatment (nb. some studies include active surveillance patients) |
| **Advanced disease** | Cancer has metastasised and therefore further treatment/action possible. |
| **Multi- stage** | Not a part of pathway but some studies include patients with prostate cancer at more than one stage of treatment pathway |
