# Supplementary material for: Supportive care for men with prostate cancer: why are the trials not working? A systematic review and recommendations for future trials
Source: Cancer Med. 2015 Apr 1;4(8):1240–51. doi: 10.1002/cam4.446 (PMC4559035; doi:10.1002/cam4.446)

**Figure 2:** forest plots summarising available data from trials with outcomes of QoL

1. Various QoL up to 12 months


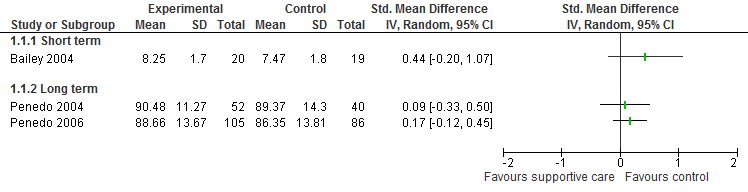


1. SF-36 MCS 2 weeks


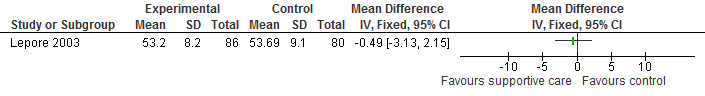


1. SF-36 PCS 2 weeks


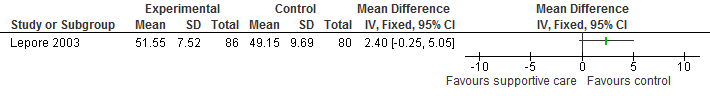


1. SF36 MCS 6 week to 6 months


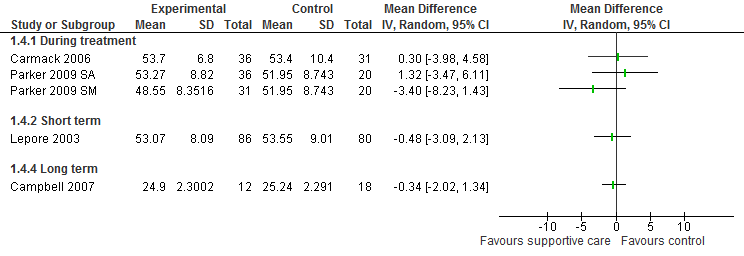


1. SF36 PCS 6 week to 6 months


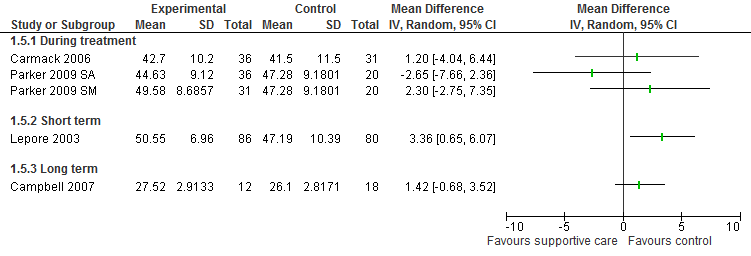


1. SF36 MCS 6 month to 12 months


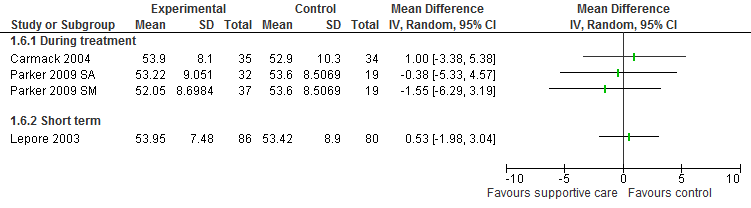


1. SF36 PCS 6 month to 12 months


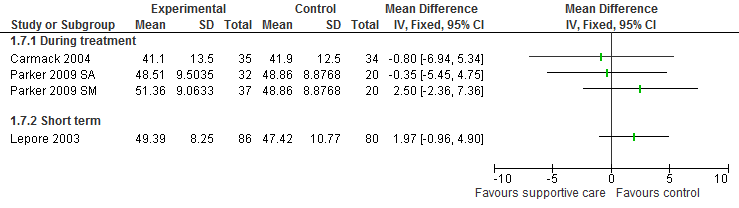

Supplement: Supplementary file 4 [file cam40004-1240-sd4.docx]
