# Supplementary material for: Supportive care for men with prostate cancer: why are the trials not working? A systematic review and recommendations for future trials
Source: Cancer Med. 2015 Apr 1;4(8):1240–51. doi: 10.1002/cam4.446 (PMC4559035; doi:10.1002/cam4.446)
Supplement: Supplementary file 5 [file cam40004-1240-sd5.docx]

**Figure 3:** forest plots summarising available data from trials with outcomes of depressive symptoms, mood and anxiety

1. **Depressive symptoms**

**
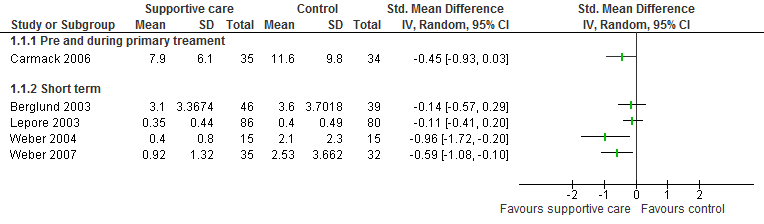
**

1. **Mood**

**
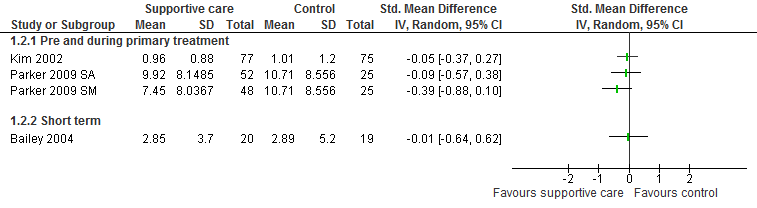
**

1. **Anxiety**

**
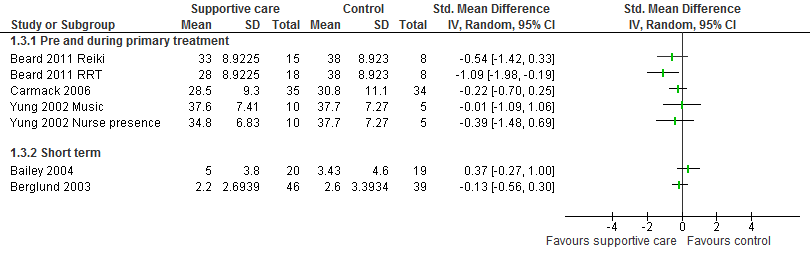
**
