# Supplementary material for: Supportive care for men with prostate cancer: why are the trials not working? A systematic review and recommendations for future trials
Source: Cancer Med. 2015 Apr 1;4(8):1240–51. doi: 10.1002/cam4.446 (PMC4559035; doi:10.1002/cam4.446)
Supplement: Supplementary file 6 [file cam40004-1240-sd6.docx]

**Table 1a-d:** Risk of bias tables for all studies

A: Studies of interventions conducted pre and during primary treatment (n=12)

| **Study Name: Beard 2011 [13]** | | |
| --- | --- | --- |
| **Bias** | **Authors' judgement** | **Support for judgement (Type in here the reason you have attributed this risk)** |
| Random sequence generation (selection bias) | Low risk | Subjects were randomized before starting EBRx using a  computer-generated randomization list derived from permuted  blocks in equal proportions to the Reiki, RRT/CR,  or wait-list control groups.-wow |
| Allocation concealment (selection bias) | Unclear risk | No detail |
| Blinding of participants and personnel (performance bias) | High Risk | Not possible but were given the option of active treatments at the end. |
| Blinding of outcome assessment (detection bias) Subjective | Unclear risk | No detail |
| Incomplete outcome data (attrition bias) | Low Risk | Detailed flow of patients and low dropouts |

.

| **Study Name: Carmack-Taylor 2004/2006/2007 [14-16]** | | | | | | | | | |
| --- | --- | --- | --- | --- | --- | --- | --- | --- | --- |
| **Bias** | | **Authors' judgement** | | | **Support for judgement (Type in here the reason you have attributed this risk)** | | | | |
| Random sequence generation (selection bias) | | Unclear risk | | | Participants were assigned to the study conditions using minimization using baseline scores of BMI, Time of androgen ablation therapy and use of psychotropic medication. | | | | |
| Allocation concealment (selection bias) | | Unclear risk | | | No details | | | | |
| Blinding of participants and personnel (performance bias) | | High Risk | | | No possible | | | | |
| Blinding of outcome assessment (detection bias) Subjective | | Unclear risk | | | No details | | | | |
| Incomplete outcome data (attrition bias) | | Low Risk | | | Well-reported but no description of how they dealt with it but all data ns | | | | |
| **Study Name: Johnson 1989 1987 [17,18]** | | | | | | | | | |
| **Bias** | | **Authors' judgement** | | | **Support for judgement (Type in here the reason you have attributed this risk)** | | | | |
| Random sequence generation (selection bias) | | Unclear risk | | | No | | | | |
| Allocation concealment (selection bias) | | Unclear risk | | | No | | | | |
| Blinding of participants and personnel (performance bias) | | High Risk | | | Not possible | | | | |
| Blinding of outcome assessment (detection bias) Subjective | | Unclear risk | | | Yes , “ the person who collected the data on outcomes did not know the patients group assignment.” | | | | |
| Incomplete outcome data (attrition bias) | | Low Risk | | | All outcome measures reported in results | | | | |
| **Study Name: Johnson 1996 [19]** | | | | | | | | | |
| **Bias** | | | **Authors' judgement** | | | | | **Support for judgement (Type in here the reason you have attributed this risk)** | |
| Random sequence generation (selection bias) | | | Unclear risk | | | | | No  Patients were randomly assigned to intervention groups in blocks of three separately for those who  planned to work and those not planning to work while receiving RT. | |
| Allocation concealment (selection bias) | | | Unclear risk | | | | | No | |
| Blinding of participants and personnel (performance bias) | | | High Risk | | | | | Not possible | |
| Blinding of outcome assessment (detection bias) Subjective | | | Unclear risk | | | | | One research assistant administered the information  messages, and another, blinded to patients’  group assignments, collected the data. | |
| Incomplete outcome data (attrition bias) | | | Low Risk | | | | | All outcomes were reported in results | |
| **Study Name: Kim 2002 [20]** | | | | | | | | | |
| **Bias** | | | **Authors' judgement** | | | | **Support for judgement (Type in here the reason you have attributed this risk)** | | |
| Random sequence generation (selection bias) | | | Unclear risk | | | | No detail | | |
| Allocation concealment (selection bias) | | | Unclear risk | | | | No detail | | |
| Blinding of participants and personnel (performance bias) | | | High Risk | | | | Not possible | | |
| Blinding of outcome assessment (detection bias) Subjective | | | Unclear risk | | | | No details | | |
| Incomplete outcome data (attrition bias) | | | Low Risk | | | | All outcome measures reported in results | | |
| **Loiselle** 2**010 [21]** | | | | | | | | |  |
| **Bias** | **Authors' judgement** | | | **Support for judgement (Type in here the reason you have attributed this risk)** | | | | |  |
| Random sequence generation (selection bias) | High Risk | | | Study was non-randomised | | | | |  |
| Allocation concealment (selection bias) | High Risk | | | N/A | | | | |  |
| Blinding of participants and personnel (performance bias) | High Risk | | | None mentioned | | | | |  |
| Blinding of outcome assessment (detection bias) Subjective | High Risk | | | None mentioned | | | | |  |
| Incomplete outcome data (attrition bias) | High Risk | | | Authors stated that 93% completed and that participants with missing data were excluded from analysis | | | | |  |
| **Study Name: Mishel 2002/2003 [22,23]** | | | | | | | | |  |
| **Bias** | | | **Authors' judgement** | | | **Support for judgement (Type in here the reason you have attributed this risk)** | | |  |
| Random sequence generation (selection bias) | | | Unclear risk | | | The design for the study was a 3 _ 2 randomized  block, repeated-measures design with three levels of  the intervention (uncertainty management direct, uncertainty  management supplemented, and control)  crossed with two levels of ethnicity (Caucasian and  African American). Patients were blocked on ethnicity  and randomly assigned to one of the treatment groups  or to the control group. | | |  |
| Allocation concealment (selection bias) | | | Unclear risk | | | No | | |  |
| Blinding of participants and personnel (performance bias) | | | High Risk | | | Not possible | | |  |
| Blinding of outcome assessment (detection bias) Subjective | | | Unclear risk | | | No | | |  |
| Incomplete outcome data (attrition bias) | | | High Risk | | | N  Umber randomised to each group not given. Data on dropouts not clearly described. No methods for dealing with missing data | | |  |
| **Study Name: Parker 2009,2011 [24-26]** | | | | | | | | |  |
| **Bias** | | | **Authors' judgement** | | | **Support for judgement (Type in here the reason you have attributed this risk)** | | |  |
| Random sequence generation (selection bias) | | | Unclear risk | | | No real details other than saying it was minimisation randomisation | | |  |
| Allocation concealment (selection bias) | | | Unclear risk | | | No details | | |  |
| Blinding of participants and personnel (performance bias) | | | High Risk | | | No details | | |  |
| Blinding of outcome assessment (detection bias) Subjective | | | Unclear risk | | | No details | | |  |
| Incomplete outcome data (attrition bias) | | | Unclear Risk | | | Described attrition and as per protocol analysis | | |  |

| **Study Name: Templeton** 2004 [27] | | |
| --- | --- | --- |
| **Bias** | **Authors' judgement** | **Support for judgement (Type in here the reason you have attributed this risk)** |
| Random sequence generation (selection bias) | Unclear | Using random numbers table |
| Allocation concealment (selection bias) | Unclear | Not described |
| Blinding of participants and personnel (performance bias) | High risk | It is not possible to blind participants to this intervention |
| Blinding of outcome assessment (detection bias) Subjective | High risk | It is not possible to blind participants to this intervention and all outcomes are self report |
| Incomplete outcome data (attrition bias) | High | Not described |
| Outcome reporting bias | Low | Authors appear to report all outcomes |

| **Study Name: Scura 2004 [28]** | | | | | |
| --- | --- | --- | --- | --- | --- |
| **Bias** | **Authors' judgement** | | **Support for judgement (Type in here the reason you have attributed this risk)** | | |
| Random sequence generation (selection bias) | Unclear risk | |  | | |
| Allocation concealment (selection bias) | Unclear risk | |  | | |
| Blinding of participants and personnel (performance bias) | High Risk | |  | | |
| Blinding of outcome assessment (detection bias) Subjective | Unclear risk | |  | | |
| Incomplete outcome data (attrition bias) | Low Risk | |  | | |
| **Study Name: Walker 2013 [29]** | | | | | |
| **Bias** | | **Authors' judgement** | | | **Support for judgement (Type in here the reason you have attributed this risk)** |
| Random sequence generation (selection bias) | | Unclear risk | | | No detail |
| Allocation concealment (selection bias) | | Unclear risk | | | No detail |
| Blinding of participants and personnel (performance bias) | | High Risk | | | Not possible |
| Blinding of outcome assessment (detection bias) Subjective | | Unclear risk | | | No details |
| Incomplete outcome data (attrition bias) | | Low Risk | | | Outcomes measures in methods was reported in results including sexual activity which was only mentioned in analysis section of methods |
| **Study Name: Yung 2002 [30]** | | | | | |
| **Bias** | | **Authors' judgement** | | **Support for judgement (Type in here the reason you have attributed this risk)** | |
| Random sequence generation (selection bias) | | Unclear risk | | No details | |
| Allocation concealment (selection bias) | | Unclear risk | | No details | |
| Blinding of participants and personnel (performance bias) | | High Risk | | Not sure | |
| Blinding of outcome assessment (detection bias) Subjective | | Unclear risk | | No details | |
| Incomplete outcome data (attrition bias) | | Low Risk | | All data there. | |

.

**B:** Studies of interventions conducted in the short-term (≤ 6months) post primary treatment (n=8)

| **Study Name: Bailey 2004 [31]** | | | | |
| --- | --- | --- | --- | --- |
| **Bias** | | **Authors' judgement** | | **Support for judgement (Type in here the reason you have attributed this risk)** |
| Random sequence generation (selection bias) | | Low risk | | “A table of random numbers was used to assign men to either experimental or control “ Page 341 col 1 para 1 |
| Allocation concealment (selection bias) | | Unclear risk | | No detail |
| Blinding of participants and personnel (performance bias) | | Unclear Risk | | Not mentioned |
| Blinding of outcome assessment (detection bias) Subjective | | Unclear risk | | No detail |
| Incomplete outcome data (attrition bias) | | Low Risk | | Very few drop outs – one from each group. Page 341 col 1 para 1. |
| **Study Name: Berglund 2003/2007 [32,33]** | | | | |
| **Bias** | **Authors' judgement** | | **Support for judgement (Type in here the reason you have attributed this risk)** | |
| Random sequence generation (selection bias) | Unclear risk | | No detail | |
| Allocation concealment (selection bias) | Unclear risk | | No detail | |
| Blinding of participants and personnel (performance bias) | High Risk | | No possible – intervention allocation was not acceptable to some patients | |
| Blinding of outcome assessment (detection bias) Subjective | Unclear risk | | No detail | |
| Incomplete outcome data (attrition bias) | High Risk | | Yes was described (quite a lot of dropouts/incomplete data) but no attempts to deal with it | |
| **Study Name: Giesler 2005 [34]** | | | | |
| **Bias** | **Authors' judgement** | | **Support for judgement (Type in here the reason you have attributed this risk)** | |
| Random sequence generation (selection bias) | Unclear risk | | Not described | |
| Allocation concealment (selection bias) | Unclear risk | | Not described | |
| Blinding of participants and personnel (performance bias) | High Risk | | It s not possible to blind participants to this study | |
| Blinding of outcome assessment (detection bias) Subjective | Unclear risk | | Not described | |
| Incomplete outcome data (attrition bias) | Low Risk | | 14 participants dropped out of the study after randomisation. The authors state that similar numbers dropped out of intervention and control and that the reason was inconvenience. | |

| **Study Name: Lepore 1999 [35]** | | |
| --- | --- | --- |
| **Bias** | **Authors' judgement** | **Support for judgement (Type in here the reason you have attributed this risk)** |
| Random sequence generation (selection bias) | High Risk | Described as randomised bit no details on randomisation procedure |
| Allocation concealment (selection bias) | High Risk | No detail |
| Blinding of participants and personnel (performance bias) | High Risk | None mentioned |
| Blinding of outcome assessment (detection bias) Subjective | High Risk | None mentioned |
| Incomplete outcome data (attrition bias) | Unclear | Attrition described no mention of how that was dealt with in analysis |

| **Study Name:** Lepore 2003/6 [36,37] | | |
| --- | --- | --- |
| **Bias** | **Authors' judgement** | **Support for judgement (Type in here the reason you have attributed this risk)** |
| Random sequence generation (selection bias) | High Risk | No details |
| Allocation concealment (selection bias) | High Risk | No details |
| Blinding of participants and personnel (performance bias) | High Risk | Participants not blinded (not possible) but interviewers were blind to experimental condition at baseline & did not participate in the interventions. Speakers/facilitators could not be blinded |
| Blinding of outcome assessment (detection bias) Subjective | Unclear risk | Interviewers were blind to experimental condition at baseline & did not participate in the interventions |
| Incomplete outcome data (attrition bias) | Low Risk | Attrition reported & ITT analysis, 10% lost to follow up |
| **Study Name: Manne 2011[38]** | | |
| **Bias** | **Authors' judgement** | **Support for judgement (Type in here the reason you have attributed this risk)** |
| Random sequence generation (selection bias) | Unclear risk | No details |
| Allocation concealment (selection bias) | Unclear risk | No details |
| Blinding of participants and personnel (performance bias) | High Risk | Not possible |
| Blinding of outcome assessment (detection bias) Subjective | Unclear risk | No mention |
| Incomplete outcome data (attrition bias) | Low Risk | ITT analysis, flow chart and dropouts described with reasons |
| **Study Name: Weber 2004 [39]** | | |
| **Bias** | **Authors' judgement** | **Support for judgement (Type in here the reason you have attributed this risk)** |
| Random sequence generation (selection bias) | Unclear risk | No detail |
| Allocation concealment (selection bias) | Unclear risk | No detail |
| Blinding of participants and personnel (performance bias) | High Risk | Not possible |
| Blinding of outcome assessment (detection bias) Subjective | Unclear risk | No detail |
| Incomplete outcome data (attrition bias) | Unclear risk | 2 dropped out before trial started , rest completed |
| **Study Name: Weber 2007 [40]** | | |
| **Bias** | **Authors' judgement** | **Support for judgement (Type in here the reason you have attributed this risk)** |
| Random sequence generation (selection bias) | Unclear risk | No detail |
| Allocation concealment (selection bias) | Unclear risk | No detail |
| Blinding of participants and personnel (performance bias) | High Risk | No detail |
| Blinding of outcome assessment (detection bias) Subjective | Unclear risk | No detail |
| Incomplete outcome data (attrition bias) | High Risk | Attrition given |

**C:** Studies of interventions conducted in the longer term (>6mths) post primary treatment

| **Study Name: Campbell** 2010 [41] | | |
| --- | --- | --- |
| **Bias** | **Authors' judgement** | **Support for judgement (Type in here the reason you have attributed this risk)** |
| Random sequence generation (selection bias) | High Risk | No detail |
| Allocation concealment (selection bias) | Nigh Risk | No detail |
| Blinding of participants and personnel (performance bias) | High Risk | No detail |
| Blinding of outcome assessment (detection bias) Subjective | High Risk | No detail |
| Incomplete outcome data (attrition bias) | High Risk | Yes, given 15 of the 20 couple completed intervention of the 15, two provided pre-treatment data only, 1 couple ‘s data was considered outlying & was excluded  Control grp , 2 couple provide pre-treatment data only- n=18 used |

.

| **Study Name: Molton 2008 [42]** | | |
| --- | --- | --- |
| **Bias** | **Authors' judgement** | **Support for judgement (Type in here the reason you have attributed this risk)** |
| Random sequence generation (selection bias) | Unclear risk | The authors describe 3 randomisation procedures and for ONE of which ONLY the generation of the sequence is described as ‘Flipping a coin’. This method was not used for one of the other two methods though as these had ratios of 2 experimental to 1 control. Page 530 col 2 para 2. |
| Allocation concealment (selection bias) | Unclear risk | Not described. |
| Blinding of participants and personnel (performance bias) | High Risk | It s not possible to blind participants to this study |
| Blinding of outcome assessment (detection bias) Subjective | Unclear risk | Not described. |
| Incomplete outcome data (attrition bias) | High Risk | 20/121 men dropped out of the study . But no information about why they dropped out. Authors performed a completers analysis with only those with data at follow up included. No Information about handling missing data. |

| **Study Name: Penedo 2004 [43]** | | |
| --- | --- | --- |
| **Bias** | **Authors' judgement** | **Support for judgement (Type in here the reason you have attributed this risk)** |
| Random sequence generation (selection bias) | Unclear risk | The authors randomised in three cohorts. In the third groups were randomised by flipping a coin’ sequence generation not described for other 2 cohorts. (Page 195 col 1 para 2). Unclear risk because not described in full. |
| Allocation concealment (selection bias) | Unclear risk | Not described |
| Blinding of participants and personnel (performance bias) | High Risk | It s not possible to blind participants to this study |
| Blinding of outcome assessment (detection bias) Subjective | Unclear risk | Not described |
| Incomplete outcome data (attrition bias) | Unclear Risk | Attrition for the 537 Penedo 2004 paper not described. |

| **Study Name: Penedo 2006 [44]** | | |
| --- | --- | --- |
| **Bias** | **Authors' judgement** | **Support for judgement (Type in here the reason you have attributed this risk)** |
| Random sequence generation (selection bias) | Unclear risk | The authors describe 3 randomisation procedures and for ONE of which ONLY the generation of the sequence is described as ‘Flipping a coin’. This method was not used for one of the other two methods though as these had ratios of 2 experimental to 1 control. Page 263 col 2 para 1. |
| Allocation concealment (selection bias) | Unclear risk | Not described. |
| Blinding of participants and personnel (performance bias) | High Risk | It s not possible to blind participants to this study |
| Blinding of outcome assessment (detection bias) Subjective | Unclear risk | Not described. |
| Incomplete outcome data (attrition bias) | High Risk | Attrition is not well described; the consort diagram and the text do not tally. Fig 1 indicated 26 men dropped out of the intervention arm and 16 from the control arm with: intervention N=107 & control N=84. But in the text on page 266 they state that at follow up there were data for intervention n=105 and control n=86. More dropped out of intervention 21% than control 14%, Authors performed a completers analysis with only those with data at follow up included. |

| **Study Name: Traeger 2013 [45]** | | |
| --- | --- | --- |
| **Bias** | **Authors' judgement** | **Support for judgement (Type in here the reason you have attributed this risk)** |
| Random sequence generation (selection bias) | Unclear risk | Not described |
| Allocation concealment (selection bias) | Unclear risk | Not described |
| Blinding of participants and personnel (performance bias) | High Risk | It is not possible to blind participants to this study |
| Blinding of outcome assessment (detection bias) Subjective | Unclear risk | Not described |
| Incomplete outcome data (attrition bias) | Unclear Risk | Similar proportion dropped out of both arms of the study. No explanation for those who dropped out was given. |

**D:** Studies of interventions delivered to men at any stage of their cancer/treatment (n=1)

| **Study Name: Northouse** 2007 [46] | | |
| --- | --- | --- |
| **Bias** | **Authors' judgement** | **Support for judgement (Type in here the reason you have attributed this risk)** |
| Random sequence generation (selection bias) | Unclear | Not described |
| Allocation concealment (selection bias) | Unclear | Not described |
| Blinding of participants and personnel (performance bias) | High Risk | Participants not blinded but research personal (data collection nurses) were |
| Blinding of outcome assessment (detection bias) Subjective | Low risk | YES – see above |
| Incomplete outcome data (attrition bias) | Unclear | Attrition described- effect on results unclear |
