# Supplementary material for: Supportive care for men with prostate cancer: why are the trials not working? A systematic review and recommendations for future trials
Source: Cancer Med. 2015 Apr 1;4(8):1240–51. doi: 10.1002/cam4.446 (PMC4559035; doi:10.1002/cam4.446)
Supplement: Supplementary file 7 [file cam40004-1240-sd7.docx]

**Table 2a:** Supportive care interventions for men with prostate cancer for pre and during primary treatment

| **Study details and Participants**  1.Study type, location  2.Baseline characteristics  3.Eligibility criteria  4.Funding  5 Power calculation  6.Risk of bias* ○=Unclear risk ✓=Low risk 🞫=High risk | **Intervention group(s)** | **Key outcome measures** 1.Relevant outcome measures  2.Follow-up  3.Attrition | **Authors’ results** |
| --- | --- | --- | --- |
| **Beard 2011 [13]**  **1.**  Pilot RCT  Three arm. n= 134 **Participants**: men receiving EBRx for prostate cancer  Boston, USA  **2.**  **Median & age range:** for Reiki 62 [50-77] RRT 66 [46-91]& control 65 [56-77]  **Ethnicity:** 91% white  **Treatment:** Reiki vs. RRT vs. Control No **(%)** RT without HT 2 (11) 1 (6) 2 (11) RT with HT 16 (89) 17 (94) 16 (89) Prior prostatectomy 3 (17) 2 (11) 3 (17) Overall 91% on HT  **Marital status:** 82%  **3.**  Male patients ≥30 years Had a biopsy-proven diagnosis of non-metastatic PC were not receiving chemotherapy  Had elected to receive EBRx  **4.**  Advanced  Medical Research Foundation, Boston, Massachusetts.  **5.**  No  **6.**  **✓○🞫○✓** | **Intervention 1:**  Reiki therapy **n=18**  **Delivered to:** Men  **Delivered by:** 1-3 experienced, nurse master Reiki practitioners  **Intensity:** Twice weekly for 8 weeks, total 16 sessions.  **Individualised therapy?:** No  **Procedure**  Reiki delivered before EBRx (same day).  **Components of therapy**  **1.**Reiki  **2.** Intervention delivered to individual  **Intervention 2**  Relaxation response therapy (RRT) & Cognitive reframing (CR)  **n=18**  **Delivered to:** Men  **Delivered by:** A psychologist  **Intensity:** weekly for 8 weeks, total of 8 sessions.  **Individualised therapy?:** No  **Procedure**  RRT delivered before EBRx (same day). Participants encouraged to practice RRT/CR techniques daily between sessions & to document these in home journal.  **Components of therapy**  **1.**Relaxation therapy  **2.**Psychological approach – cognitive restructuring  **3.** Intervention delivered to individual.  4. Homework  **Control**  Wait-list  **n=18**  Either Reiki or RRT/CR at end of study. 2/18 elected to receive Reiki after completing study. | **1.**  **Anxiety**  STAI- 20 item  **Depression**  CES-D- 20 item  **Quality of life**  FACT-G-27 item  **2.**  At 0, 4, 8 20 weeks  **3.**  RRT group: 16/18 (88%) & R group: 15/18 (83%) attended all sessions.  Psychological & physiological testing was completed by 80-100% & 86% completed follow-up assessment. | **Anxiety**  STAI- 20 item  No difference between interventions and controls  **Depression**  CES-D- 20 item  No difference between interventions and controls  **Quality of life**  FACT-G-27 item  No difference between interventions and controls  But Emotional wellbeing sub-scale: Participants who received the RRT intervention had better Emotional wellbeing than those in Reiki (p=0.02) or control (p=0.01) |
| **Carmack Taylor** 2004,2006,2007 [14-16]  **1.**  RCT  Three arm  134 men receiving HT  Houston  USA    **2.**  **Mean age:** (range 44.8–89.0)  **Ethnicity:** 73.1% white, 20.1% Black/African American, 6.7% other  **Treatment:** participants had been on androgen ablation therapy an average of 32.7 months 12 (9%) by orchiectomy 122 (91%) LHRH agonist injections  **Marital status:** 82.8% married/living with a significant other  **Plus: ~**34% had  one medical condition in addition to prostate  cancer e.g. hypertension, heart conditions, diabetes, rheumatoid arthritis& respiratory problems ~14% reported current use of psychotropic medication.  **3.**  Participants with PC  regardless of stage,  Receiving continuous androgen-ablation therapy with expectation to continue therapy for ≥1 year. Residing within 1 h of The University of Texas Anderson Cancer Centre. To be able to provide informed consent. To be able to speak & understand English.  **4.** American Cancer Society  **5.** Yes  **6.○○🞫○✓** | **Intervention 1:** Active for life (lifestyle program). **n=46**  **Delivered to:** Men  **Delivered by:** Group facilitators supervised by a licensed clinical psychologist  Physical therapist (exercise). Expert speakers (sexuality, treatment, side effects or diet)  **Intensity:** 6 months duration with one orientation session, 16 weekly sessions and four biweekly sessions -all 1.5 hrs. long.  **Individualised therapy?:** No  **Procedure:** 60 mins of CBT curriculum focused on increasing physical activity. No physical activity skills training were provided. 30 mins of a facilitated discussion or an expert speaker covering topics such sexuality, treatment side effects, or diet. Incentives for session attendances & homework plus intermittent door prizes (being on time). Intervention also included diary keeping, monitoring and goal setting.  **Components of therapy**  1**.**Education  2.Psychological approach :CBT for physical activity  3. Intervention delivered to group  4.Peer discussion  5. Homework  **Intervention 2:** Educational support program. **n=50**  **Intervention delivered to:** Men  **Intervention delivered by:** Group facilitators supervised by a licensed clinical psychologist, Expert speakers (sexuality, treatment, side effects or diet)  **Intensity:** as above  **Individualised therapy?:** No  **Procedure:** 60 mins of facilitated discussion. 30 mins of either a facilitated discussion or an expert speaker covering topics such as sexuality, treatment side effects, or diet. Incentives for session attendance plus arriving on time (randomly)  **Components of therapy**  1.Education  2.Intervention delivered to group  **Control:** ‘Standard care’ **n=38**  All groups received written educational & informational material. | **1.**  **QoL**  SF-36  **Depression**  CES-D  **Anxiety**  STAI  **Pain**  BPI  **Social support**  Interpersonal support Evaluation List  **2.**  At baseline, 6 & 12 months follow up  **3.**  Well described -19 lost during interventions | **1.**  **QoL**  SF-36 No statistically significant difference between intervention and control groups  **Depression**  CES-D  No statistically significant difference between intervention and control groups  **Anxiety**  STAI  No statistically significant difference between intervention and control groups  **Pain**  BPI  **Social support**  *Authors report:*  The lifestyle approach is a promising means for promoting adoption and adherence for some individuals; however, our data suggest that cognitive-behavioural skills training alone is an insufficient strategy for getting prostate cancer patients receiving continuous androgen-ablation to adopt routine lifestyle activity. |
| **Johnson** 1988/9 [17,18]  **1.**  RCT  Two arm  84 men  Rochester  USA  **2.**  **Mean age:** 67.9yrs (range 50-85yrs)  **Ethnicity:** 96% White  **Treatment:** about to receive RT for first time  **Marital status:** 83% married  **3.**  PC patients who were about to receive RT as outpatients. No prior RT. Free of other cancers. Able to speak & read English. Cognitively oriented. Capable of meeting daily basic needs independently & give written informed consent.  **4.**  National Cancer Institute & Robert Wood Johnson Foundation  **5.**  No  **6.**  **○○🞫○✓** | **Intervention 1:** Informational intervention with descriptions of the experience on outcomes of coping with radiation therapy  **n=42**  **Intervention delivered to:** Men about to undergo RT  **Intervention delivered by:** Research staff member who stayed with patients while the tape recordings were played or engaged comparison group patients in conversation.  **Intensity:** Immediately prior to treatment planning session, prior to first treatment, at fifth treatment & during last week of treatment **Individualised therapy?:**No  **Procedure:** 30 min prior to treatment, men gave consent, completed POMS, & randomized & received 1^st^ tape recorded message (treatment planning process & immediately) 2^nd^ message: delivered prior to 1^st^ treatment (size & location of treatment room , sound of machine & length of treatment) 3rd message delivered at time of 5^th^ treatment (nature, timing & pattern of side effects following treatment) 4^th^ message: delivered during last week of treatment (description of changes in side effects following treatment completion). The last two messages also contained self-care advice.  **Intensity:** unclear  **Components of therapy**   1. Information 2. Intervention delivered to individual   **Control:** **n=42**  Patients contacted by same research staff member & with general inquiry about well-being & social conversation. Specifics about RT experience were avoided; if the patients requested such information, they were asked to consult their physician/nurse. | **1.**  **Coping** (recreation & pastime subscale of the Sickness Impact Profile  **Mood**  (POMS 5 item)  2.  At time of 3rd treatment. (5 -10 days after treatment planning) & 3^rd^ & last weeks of treatments.  During treatment data & 1 month post treatment data were collected by interview. At follow-up appointments, data collected by mail or telephone. 3-month data obtained by mail, phone & interview.  **3.**  97 men judged to be eligible, 95 agreed to participate. 11 not included in analyses because of withdrawal n=3, abnormal treatment planning experience n =1, or not meeting eligibility criteria after entering study (cognitive or language deficits, n = 4; presence of metastatic disease, n = 3. | 1 **Coping**  One subscale indicated both groups improved over time (Recreation and pastimes subscale)  F(4,328)=0.833 p<0.001  And the intervention group improved greater than the controls F(1,82)=6.57 p<0.02  **MOOD POMS**  There were no differences between the intervention and control.  The level of negative mood reported by these older men who had an excellent prognosis was relatively low at each of the occurrences of measurement.  *Authors report*  The experimental group had significantly less disruption in function during and for 3 mths following RT than the comparison group. The intervention had no significant effect negative mood |
| **Johnson** 1996 [19]  **1.**  RCT  Three arm  Patients randomly assigned to intervention  groups in blocks of 3 for those who planned to work & those not planning to work while receiving RT.  62 men  Rochester  USA  2.  **Mean age:** 69.6yrs SD 6.9  **Ethnicity:** 97% White **Treatment:** 68% stage B, rest were Stages A & C  **Marital status** 84% were married  **3.**  Patients scheduled to receive RT as outpatient for treatment of localized PC. No previous or other concurrent cancer diagnosis except basal cell skin cancer. No history of treatment for a mental condition within past 5 years. Comprehension of written & spoken English. Able to carry on normal activities, cognitively able to fill in questionnaires and ≥18 years old.  **4.**  American Cancer Society and for the write up National Centre of Nursing Research to main author, and a grant from National Cancer Institute to another author.  **5.**  No  **6.**  **○○🞫○✓** | **Intervention 1**  Coping group  **n=22**  **Intervention delivered to:** Patients scheduled to receive RT as an outpatient for treatment of localized PC  **Intervention delivered by:** One research assistant  **Intensity:** 3 audiotaped messages during RT treatment (time period unclear)  **Individualised therapy?:** No  **Procedure:** Patients received written summary of information covered each message. The purpose of providing information was included in first message for each group & repeated briefly in messages 2 & 3. The stated purpose was coping and self-care.  **Intensity:** unclear, during RT  **Components of therapy**  1.information  2.Intervention delivered to individual  **Intervention 2**  Concrete group  **n=20**  **Intervention delivered to:** Patients scheduled to receive RT as an outpatient for treatment of localized PC  **Intervention delivered by:** One research assistant  **Intensity:** Three audiotaped messages during RT treatment (time period unclear)  **Individualised therapy?:** No  **Procedure:** Patients received written summary of information of each message. The purpose of providing information was included in first message for each group  and repeated briefly in messages 2 & 3. The  stated purpose were concrete objectives to allow the patient to know what to expect and understand what would happen, which would help him to deal with the experience of receiving RT.  **Intensity:** unclear, during RT  **Components of therapy**  1.Information  2.Intervention delivered to individual  **Control: n=20**  Patients received written summary information from each message.  The purpose of providing information  was included in the 1st message for each group  & repeated briefly in messages 2 & 3*.* The purposes were to provide information about therapy and services provided by Cancer Centre. | **1.**  **Mood**  (POMS-BI -72 )  **Coping**  (Sickness Impact Profile- five categories’ recreation & pastimes, sleep-rest, home management, mobility & social interaction)  Patients were stratified into optimistic or pessimistic using the Life Orientation Test (LOT eight items)  **2.**  The 2-week post-RT data collected for majority by telephone. Remaining patients responded in clinic n = 2 or by mail n = 4. The one-month post-RT data were collected in clinic, by telephone & by mail. At 3 months, data were collected in clinic, by telephone & by mail.  **3.**  21 eligible patients (23%) refused to participate; the amount of time required was most frequent reason. 2 control group patients withdrew. Three patients were dropped from  study, n=1 with a severe complication  of RT (coping group) and 2 as a result of  health problems during RT (1, control; 1, concrete group | **1 Mood**  Mood (POMS)-BI-72 item. No difference between groups for Mood scores.  Post-hoc subgroup analysis: The mean mood scores of men assigned to the ‘Concrete’ group was improved compared with the mood of low-optimism men in the control and other intervention groups t(3,53)=2.22, p<0.05)  **2 Coping**  Disruption of usual activities (Sickness Impact Profile) There were no differences between the intervention groups. |
| **Kim** 2002 [20]  **1.**  RCT  Two arm  184 men receiving RT from 8 cancer centres were enrolled in (91-97)  Rochester  USA  **2.**  **Mean age:** 70.8 years (range 44–85 years).  **Ethnicity:** Caucasian (96%)  **Disease status:** 13% stage A, 66% stage B, & 21% stage C disease. Most of patients (92%) did not receive hormone therapy (previously?)  **Marital status:** Married 86%.  **3.**  Receiving RT as curative treatment for localized PC as outpatients. No previous or concurrent cancer diagnosis (except basal cell skin cancer).  Able to speak and read English. No history of mental illness or alcoholism. Capable of meeting daily basic needs independently. Karnofsky Performance status 80%) and ≥18 years old.  **4.**  National Cancer Institute  **5.**No  **6.**  **○○🞫○✓** | Informational intervention  **n=77**  **Intervention delivered to:** Men about to undergo RT  **Intervention delivered by:** Research staff member – no further details.  **Intensity:** 2-tape recorded messages before their 1^st^ & 5^th^ RT (4 & 8 minutes, respectively)  **Individualised therapy?:** No  **Procedure:** Patients listened to brief tape-recorded messages in clinic before 1st & 5th RT sessions. A member of research staff stayed with each patient while tape recordings were played. The first tape described clinical set-up, & what would occur at 1st treatment.  The 2^nd^ tape described what would occur during the succeeding weeks of treatment.  The messages focused on physical & sensory experiences associated treatment (e.g. buzzing sound as the machine moved); environmental characteristics (e.g., size of treatment room); & temporal characteristics (e.g., when specific side effects most likely to occur).  **Components of therapy**  1. information  2.intervention delivered to individual  **Control:**  **n=75**  As for intervention but content of tape recorded messages was different & were about general & global information generally available to all RT patients, including resources available. | **1.**  **Mood-negative affect** (the tension-anxiety, anger-hostility & depression-dejection subscales of POMS)  [also collected severity of side effects & demographics ]  **2.**  Measured post-listening to tapes at 1^st^ & 5^th^ treatments  **3.**  Because of data  management errors, (improper randomization or lost data n=12, refusal to complete questionnaires  n=6 & incomplete data n=14, only 152 patients were fully evaluable. No differences in demographic, clinical, & study variables were found among the patients who did & did not complete. | **1. Mood:**  Mood (POMS)  There were no differences in POMS score between intervention groups F=0.10 p>0.10  Authors calculated a change in POMS score over time – when they analyses this using logistic regression analysis  These authors report that their intervention was beneficial for side effects of reducing fatigue and sleep problems resulting from treatment for cancer. |
| **Loiselle** 2010 [21]  **1.**  Pre/post controlled study  Two arm  (intervention group from 3 hospitals, controls from 4th hospital )  45 men  (plus 250 breast cancer patients)  Montreal  Canada  **2.**  **Mean age:** 65yrs  **Ethnicity:** Caucasian 80.5%  Other 19.5%  **Treatment:** no details  **Marital status:** Single 5%, married/partner 80%, separated/divorced/widowed  10%.  **3.**  Recent diagnosis (3-8wks) of prostate cancer. Accepting treatments & follow-up at study site. Fluent in English or French. Cognitively & physically capable of participating 7 completing questionnaires. No Concurrent major illness or chronic mental health problem.  **4.**  Canadian Institutes of Health Research & Fonds de la Recherche en sante´ du  Quebec.  **5.**  Yes  **6.**  🞫🞫🞫🞫🞫 | **Intervention:** Information via internet  **n=28**  **Delivered to:** Men  **Delivered by:**A medical librarian or a trained volunteer  **Intensity:** one training session & the used as required  **Individualised therapy?:** No  **Procedure:** 1-on-1 one hr IT training session on how to use internet, direction to reputable cancer websites educational CD ROM.  Patients kept IT material for 8wks to use whenever needed. IT support given during 8 wk period by email or phone  [3 patients without laptops were provided with one during study period]  **Components of therapy**  1.information-[internet training]  2. intervention delivery to individual  **Control:**  ‘Usual care’ **n=17**  The control sites were not using IT during study period. Usual care comprised of nurse navigator to provide initial cancer diagnosis (e.g. pamphlets) & oncology clinics offered diagnostic treatment, psychosocial support when needed & follow up services  Control & intervention sites were close geographically & were university affiliated. | **1.**  **Anxiety**  (STAI )  **Depression**  (CES-D)  **General QoL**  (SF-36 )  **Wellbeing**  7(Index of well-being Oncologist informational Support scale)  **Self-esteem** (Rosenberg self-esteem scale item?)  **Life orientation** (Lot-R item?)  **Sense of mastery** (SOM item?)  **Overall satisfaction with cancer treatment**  **2.**  Pre intervention, 1-2 wks post intervention & 3mth post intervention  Those not available in French were translated by PI and RA using back and forth translation  **3.**  “93% of participants completed all 3 measurement points.” No other detail. | **Anxiety STAI**  Anxiety decreased over the course of the intervention T1-T3 for Men F(2,76)=4.20 p=0.019  **Depression**  Ces-D  Men’s depression reduced between T1 and T3 (t=2.33, p=0.07)  **QoL SF36 MCS** Mental component score  No difference between intervention and control, nor over time. F(2,76) =1.89, p=0.158.  **QoL SF36 PCS** Physical and wellbeing. No differences in physical functioning over time.  The analysis was for men and women and the study was not randomised. Therefore for men – the analysis of data data are subject to confounding because they were not randomised. |
| **Mishel** 2002,2003 [22,23]  **1.**  RCT  Three arm  239 Caucasian men & 105 African-American men with localised prostate cancer  North Carolina  US  **2.**  **Mean age:** 64.0 ±6.9 years  **Ethnicity:** 56% Caucasian men,44% African-American  **Treatment:** 8% had stage T1 tumours, 61% stage T2 tumors,27% had stage T3 tumours, 4% of patients no information, 56% had RP  23% nerve sparing surgery, 21% RT  **Marital status:**84%married  **3.**  African-American men & Caucasian men diagnosed with localized prostate  cancer & were within 2 weeks post-catheter removal after undergoing surgical treatment and/or were within 3 weeks into current radiation therapy. Had a telephone & an identifiable family member willing to participate in the study & planned to reside in their current community for 12 months. No major cognitive impairment. No concurrent treatment for another form of malignancy.  **4.**  National  Cancer Institute & National Institute of Nursing  Research  **5.**  Yes  **6.**  **○○🞫○🞫** | **Intervention 1**  Psycho education intervention by telephone  **n=not given**  **Delivered to:** Men  **Delivered by:** Nurse trained in the intervention. Nurses were matched with the patient & family member by ethnicity & gender.  **Intensity:** Weekly phone calls for 8 weeks  **Individualised therapy?**  Yes  **Procedure:** During each call, nurse used a semi-structured interview to assess the patient’s concerns with a standardized list of possible problems but also encouraged patient to express his individual specific concerns. Approaches included for cognitive reframing, problem solving, and strengthening patient-provider communication promotion of self-advocacy.  **Intensity:**  Weekly phone calls for 8 weeks  **Components of therapy**   1. Psychological approach- cognitive restructuring and psycho-education including problem solving and communication skills 2. Intervention delivered to individual   **Intervention 2**  Psycho education intervention by telephone for 8 weeks.  **n=not given**  **Intervention delivered to:** Men plus delivery to a close family members  Other details as above  **Components of therapy**   1. Psychological approach – psycho-education 2. Intervention delivered to Individual   **Control:** ‘Usual care’ **n=not given**  During data collection, printed general health information was given by nurse None of the information was related to prostate cancer or the side effects from treatment. They offered men 4 intervention calls after completing data collection. | **1.**  **Uncertainty** Uncertainty in Illness (Likert) scale 5 item  **Uncertainty management** (problem solving (10 item) & cognitive reframing (9 item) subscales on Self-control scale)  **Cancer knowledge** (Cancer knowledge scale 21 items)  **Patient & provider communication** (authors own scale 5 items)  **QoL**  (Southwest oncology group quality of life questionnaire incorporating 15 item symptom distress scale)  **2.**  Measured at baseline, 4 & 7 months  **3.**  “There was drop- out rate of 5% “ – no further detail | **1 Uncertainty**  This declined with time but was no different between interventions and control group.  **Problem solving**  No difference between the groups at 7 months (F1,229 = 6.38; p=0.012)  **Cognitive reframing**  No difference between groups at 7 months  **Sexual functioning** No difference between groups at 7 months  **Uncertainty management**  Authors report the differences were significant (Wilks Lambda F16,438 = 1.96;P=0.01)  **Problem solving**  No difference between the groups at 7 months (F1,229 = 6.38; p=0.012) But control group declined in score for this from baseline to 4 months – regaining baseline values at 7 months whereas control groups steadily increased in problem solving and didn’t lose any ability at 4 months.  **Cognitive reframing**  Was similar for all groups at 7 months. P1861 col 1 para 2. Data presented for groups combined versus control. BUT Cognitive reframing declined for control at 4 months and then regained baseline levels. Intervention groups did not lose cognitive reframing at 4 months.  **Sexual functioning &** sexual satisfaction was improved at 4 mths compared with control in some sub-analysis |
| **Parker** 2009 [24]  **Cohen** 2011 [25]  **Gilts** 2011 [26]  **1.**  RCT  Three arm (2 control groups)  159 men after RP  Texas  USA  **2.**  **Mean age:** 60 yrs  **Ethnicity:** 78% white, 13% African American, Hispanic Latino 4%, Asian 2%, other 3%  **Treatment:** about to have RP  **Marital status:** 85% Married/living with partner, 15% divorced/separated/  widowed/never married  **3.**  Men with early stage prostate cancer undergoing RP at 1 of 3 hospitals in Texas. Age >18yrs. Able to speak & write in English. Able to come to medical centre 4x before surgery or live with 100 miles of medical centre  Not having had other major surgery in the preceding yr or any major psychiatric diagnosis or currently undergoing psychiatric treatment or psychological counselling.  **4.**  National Institute of Mental Health & National Cancer Institute  **5.**  Yes  **6.**  ○○🞫○○ | **Intervention 1**  Two session pre-surgical stress management (SM) intervention  **n=53**  **Intervention 2**  n=54  Two session pre-surgical “supportive attention” (SA) group  **Interventions delivered to:** Men who were able to have RP surgery  **Intervention delivered by:** Both delivered by a clinical psychologist  **Intensity:** Both 2 x 60-90 minute sessions plus two brief booster sessions on the morning of surgery & 48hrs after surgery  **Individualised therapy?:**  Yes for both  **Procedure**  SM intervention was individual sessions based on a Stress Management Guide containing info on relaxation, coping skills & information about prostate cancer & RP including side effect management. During the rest of the sessions, men discussed their concerns or fears about cancer & surgery & learned problem-focused coping strategies e.g. activity pacing, seeking out social support, & having realistic expectations about recovery. Two brief booster sessions reinforced the use of relaxation strategies & problem-focused coping strategies.  **Manualised?** Yes  **Theory based?** Yes  SA sessions gave patients extra attention from medical community & provided encouraging environment to discuss their concerns. Two brief boosters in which they discussed their experiences leading up to the surgery and during their hospital stay.  **Components of SM therapy**  1.Psychological approach -CBT stress management & psycho education  **3.**Relaxation therapy  4.Intervention delivered to individual  5. Discussion with health professional  **Components of SA**  **therapy**   1. psychological approach general [attention placebo]   **Control:** Standard care (SC) or ‘Routine medical care’  **n=52**  Patients in the standard group had no meetings with a clinical psychologist. | **1.**  **Mood disturbance** (POMS -18 item)  **General quality of life**  (SF-36-MOS)  **Prostate cancer specific quality of life**  (UCLA PCI)  [IES also measured]  Cohen {133} also described immunological outcomes.  Gilts {1678} also described a post hoc analysis relating baseline social support, distress & coping style with the QoL  **2.**  One week before surgery & morning of surgery, 6weeks, 6 months &12mths  **3.**  159 men were randomized & 150 men completed the POMS at each time point (SM - 48; SA - 52; SC -50). | 1. Mood POMS  Mood was better in SM than SC group at the pre-surgery time point (1week before surgery) SM mean 10.26 n=48, SA Group mean = 9.63 n=52, SC group (control) mean = 13.13 n=50 - p=0.006) . But there were no differences in mood for the three groups over other time points (6 weeks, 6 months and 12 months post-surgery).  2. MOS-PCS was better in SM grp than SC group (p=0.0009) at 12mths  No differences between groups with UCLA-PC or MOS-MCS  Authors measured the Impact of events scale – (Intrusively experienced ideas or feelings – intrusive thoughts) The scores were similar for all groups at all time points.  Gilts 2011 {1678} post hoc analysis suggests distressed individuals may respond more from unstructured discussion about distress and those with low social support may benefit from a more structured coping skills approach |
| **Templeton** 2004 [27]  **1.**  RCT  Two arm  Londonderry  Northern Ireland  Two centres  58 men with PC on HT recruited from  two urology centres in  Northern Ireland  **2.**.  **Ages:** 51-60yrs 12.65%,61-70yrs 29.0%, 71-80yrs 41.9%**,** 80+yrs 16.4%  **Marital status:** Married 69%,Single/divorced/widowed /separated 31%  No other details  **3.**  Men with a known diagnosis of prostate cancer, who commenced HMT within the year 2000. But not confused or terminally ill patients & patients who were unaware of their diagnosis.  **4.**  Mona Grey Research Scholarship, Astra Zeneca Pharmaceuticals and the Prostate Research Campaign, UK.  **5.**  No  **6.**  ○○🞫🞫🞫 | An evidence-based education package  **n=29**  **Delivered to:** Men who had commenced HMT  **Delivered by:** Urology nurse  **Intensity:** Not clear.  **Individualised therapy?:** Yes  **Procedure**  A structured interview conducted in participants’ own homes. One month later, they received 2nd questionnaire by post. The education package was delivered to group following completion of the 1^st^ questionnaire. No detail  **Components of therapy**  **1**. Education  **2**. Discussion with HP  **3**. Intervention delivered to individual.  **Control:** **n=29** No detail | **1.**  **QoL**  Physical, social /family , emotional & functioning  (FACT-P 40 items)  **Coping**  ( JCS-40)  **2.**  Pre and post intervention. time scales not clear  Text suggests one session with a pre & post questionnaire (at home and by post respectively)  **3.**  3 drop outs- not clear which group | **1 QoL FACT G**  The authors do not report an analysis of differences between the control and intervention group at follow up. They do report a difference from pre to post test for each group.  FACT-P  All subscales were significantly improved between pre and post test  Physical p=0.002  Emotional p<0.001  Social/family p=0.001  Functional p<0.001  PC specific concern 0.023  FACT G (functional ) p<0.001  FACT-P p<0.001  2. Coping  Coping JCS-40  The authors do not report an analysis of differences between the control and intervention group at follow up. They do report that both groups used similar coping styles (Problem solving more often than affective coping styles) they also report that within each group there was no difference from baseline to follow up. Control (t=-1.35; df 26 p=0.188) Education (t=-1.585; df =27; p=0.125) |
| **Scura** 2004 [28]  **1.**  Pilot RCT  Two arm  17 men diagnosed with PC within 4 weeks of study entry  New Jersey  USA  **2.**  **Mean age:** 66 (range 51-78)  **Ethnicity:** n=10 (59%) White  n= 6 (35%) African American  n=1 (6%) American Indian  **Treatment:** n=9 WW**,** n=6 no treatment **,** n=2 RT  all anatomic stages of prostate cancer  **Marital status:** n=7 Married/relationship n=10 not married/no relationship.  **3.**  English–speaking men who were informed about their PC diagnosis within previous four weeks. Not experiencing major underlying medical problems. At all stages of prostate cancer.  **4.**  ONS research foundation fellowship & Ortho biotech products LP.  **5.**  No  **6.**  **○○🞫○✓** | **Intervention group**  Telephone social support and education via a mailed resource kit  **n=7**  **Intervention delivered to:** Men  **Intervention delivered by:** One oncology research assistant  **Procedure (& intensity)** *Phase one:* 2 weeks of weekly social support phone calls. 8 weeks of weekly social support plus structured formal education phone calls  *Phase two (10wks at start of study):* Five months of twice monthly social support plus structured formal education phone calls  *Phase three* starts at 7 mths(~5mths):  Once a month social support plus structured formal education phone calls. Included a termination phase of the research assistant –subject relationship. Participants were mailed a resource kit (PC information manual developed for this study, video tapes and an audiotape)  Total duration 12 months  **Individualised therapy?:**  Yes?  **Manualised?** Yes  **Components of therapy**  1.psychological approach –psychoeducation (general)  2. Intervention delivered to individual.  **Control:** **n=10**  Men received education via mailed resource kits only | **1.**  **QoL**  (FACT-G-28)  **Symptoms** (Symptom experience scale –prostate 24)  **Erectile dysfunction** (International index of Erectile dysfunction scale 15 item)  **Relationship change scale**  **2.**  At the three phases  Baseline – at the start of the study  Phase I – to 10 weeks after the start of the study  Phase II – 10 weeks to 5 months after the start of the study  Phase III – 7 months to 12 months after start of study  **3.**  By 12mth, 85 of original consenting dyads remained & 14 dyads had dropped out. Main reason given was inconvenience. Attrition rates were similar across both groups. | The authors describe that they found a “trend toward declining physical, functional and sexual well-being measures from Phase i to Phase ii (immediately after start of treatment to 5 months) and an increase in phase iii (7 to 12 months).” and that this is consistent with research by others and fits with side effects of prostate cancer. But that it is not possible to analyse the data to compare effects across groups as the sample sizes are too small. |
| **Walker** 2013 [29]    **1.**  Pilot RCT  Two arm  27 couples? in which the men had PC & were on androgen deprivation therapy (ADT)  Participants recruited from cancer treatment centres  Calgary & Halifax, Canada.  **2.**  **Mean age:** 73yrs (range 52-85) of men (partners not recorded)  100% Caucasian  Basic demographic data for patients was collected by self-report but not reported.  **3.**  All patients had either just started, or were scheduled to  commence ADT. Couples were excluded if the patient (or partner) lacked adequate English fluency to read educational booklet, was too unwell to be able to sit for  an hour to complete the questionnaire package, or had metastatic symptoms.  **4.**  Canadian Institutes of Health Research  **5.**  No  **6.**  **○○🞫○✓** | An educational intervention designed to preserve couples’ intimacy in the face of ADT  **n=not reported**  **Delivered to:** Couples  **Delivered by:** “by a male and female team” so that attendees would understand that the program was meant to serve equally the concerns of the male patient and the female partner (in heterosexual couples).” In general, sessions were led by researchers familiar with patient and partner adaptation to ADT.  **Intensity:** Two weeks to read a booklet & one private one hour- educational review session  **Individualised therapy?:**Yes  **Procedure:** Couples were given questionnaires & instructed to complete separately, & seal them in individual envelopes before returning. Intervention involved reading a 70-page booklet entitled: Androgen Deprivation Therapy: A Guide for Prostate Cancer Patients and Their Partners in 2 week period. and focused on managing side effects that directly affected patients & the couple. Subsequently, each couple had a private one-hour educational review session to address concerns that may have arisen while reading the booklet, & issues that may not have been covered.  **Components of therapy**  1.Education  2.Intervention delivered to couple  **Control:** ‘Usual care’  **n=not reported**  (no detail) | **1.**  **Intimacy in relationships** (PAIR- 72 items)  **Dyadic adjustment** (DAS-32 item)  **2.**  Baseline & 6 months after intervention  Couples were also asked about their sexual activity in the past month  **3.**  ‘At the Calgary site, accrual & retention  was promising, although there was significant attrition in the control group’ | **1. Intimacy in relationships:** (PAIR- 72 items)  There was no differences between the groups (as rated by couples) Mean 0.09 (SD -43,17.6) p=0.544  **Dyadic adjustment** (DAS-32 item)  There was no differences between the groups (as rated by couples)Mean  0.58 (SD = -58,10) p=0.132 |
| **Yung** 2002 [30]  **1.**  RCT  Three arm (2 control groups)  n=30 men receiving  transurethral resection of the prostate (TURP)  Hong Kong  China  **2.**  **Mean age:** 67.70 yrs (range 52–80).  **Ethnicity:** 100% Chinese  **Treatment:** TURP  **Marital status:** No details  **3.**  Men waiting to undergo inpatient surgery for TURP.  No cardiac disease and no medical history of hypertension. Had not received any pre-operative sedation, which could affect vital signs or level of consciousness. Able to comprehend oral and written instructions.  **4.**  None declared  **5.**  No  **6.**  **○○🞫○✓** | Music intervention group  **n=10**  **Delivered to:** Men about to undergo TURP  **Delivered by:** “No nurse was present whilst they were listening to the music.”  **Intensity:** 20 minutes whilst waiting for surgery  **Individualised therapy?:** No  **Procedure**  Patients listened to slow rhythm soft music via an earphone for 20 minutes before surgery in a theatre holding area. They had the choice of three slow rhythm soft music tapes: Chinese slow rhythm music and Western slow rhythm music. These music tapes, chosen by the experimenters, were examined and judged by a panel of 3 musicians teaching music at colleges who agreed that the music was sedative.  **Components of therapy**   1. relaxation therapy (music) 2. Intervention delivered to individual   **-----------------------------------**  **Intervention 2**  Nurse presence group  **n=10**  **Intervention delivered to:** Men about to undergo TURP  **Intervention delivered by:** Nurses  **Intensity:** Not mentioned but assume~20 mins  **Individualised therapy?:** No  **Procedure**  Conducted in a theatre holding area  A nurse was merely present and there was minimal verbal interaction with the patient. This represented the non-specific factor of attention given by a nurse.  **Components of therapy**   1. Attention placebo 2. Intervention delivery to individual   **Control:** Usual procedure  **n=10**  No nurse or music intervention was provided | **1.**  **Anxiety**  (State portion of STAI- 20 items)  [blood pressure & heart rate was also measured ]  **2.**  Immediately before & after intervention  **3.**  No information | **Anxiety** (Chinese STAI)  No difference between groups  Mean and SD and N for all of the groups and pre, post for all interventions and control available. |

**Table 2b:** Supportive care interventions for men with prostate cancer for short term after primary treatment

| **Author**  1.Study type  2.Baseline characteristics  3.Eligibility criteria  4.Funding  5.Power calculation  6. Risk of bias* ○=Unclear risk ✓=Low risk 🞫=High risk | **Intervention group(s)** | **Data collection**  1.Relevant outcome measures  2.Follow-up  3.Attrition | **Authors’ results** |
| --- | --- | --- | --- |
| **Bailey** 2004 [31]  **1.**  Pilot RCT  2-arm  41 Men  USA  **2.**  **Age:**75 years (mean)  **Ethnicity:**  86% Caucasian 14% African American.  **Marital status:** Not stated  **Disease stage:** 97% T1 or T2  3% stage T3.  **Treatment:**  Watchful waiting (WW) for mean of 51.8 months (range 1 to 124 months (10.3 years).  **3.**  Recruited from urology practice  Men who had elected for Watchful waiting.  **4.**  None declared  **5.**  No  **6.**  **✓ ○ ○ ○✓** | Uncertainty intervention  **n= not stated**  **Delivered to:** Men  **Delivered by:** Male nurse interventionist (NI)  **Intensity:**  5 calls one, per week. Mean duration 13 mins, (range 5-26)  **Individualised therapy?** Yes  **Procedure:**  NI called patients to identify patient’s problem & assessed nature of the patient’s uncertainty related to WW. The NI used info to deliver interventions to help the patient reframe uncertainty & incorporate uncertainty into his life. Interventions were 1) probabilistic thinking, 2) incorporating uncertainty into life 3) Support to believe that future treatment would be beneficial 4) Encouraging self-monitoring and vigilance.  **Components of therapy**  **1**. psychological approach – psycho-education & cognitive restructuring  **2.** Delivered to individual.  **Control:**  ‘Usual Care’  **n= not stated**  “Access to naturally occurring sources of support” | **1.**  **Growth through uncertainty Scale** (GTUS)  39 items – 4 domains  **Profile of Mood States** – Short Form (POMS) 37 items 6  **Quality of life** measured by Cantril’s ladder.  **Cognitive reframing**:  Rosenbaum’s Self-Control Schedule (SCS).  **2.**  T1 = baseline  T2 = 10 weeks post baseline.  **3.**  During study one participants from intervention group withdrew for personal reasons. At T2, one participants in control group had initiated treatment for prostate cancer and was dropped from sample. | **Growth through uncertainty**  No difference between intervention and control at 10 weeks. F[1,37] = 2.176 p=0.149.  Cognitive reframing: Rosenbaum’s self-control schedule  No difference between intervention and control at 10 weeks. Experimental group ability to cognitively reframe dropped by 2 points: controls dropped 7.1 points (Not significant).  POMS scores were similar in the control and intervention groups F[1,37] = 1.009, p=0.322  **Quality of life** Cantril’s ladder  QoL improved for men in the intervention group cf control. QoL scores for men receiving the intervention increased by 0.75 points whereas men in the control dropped by 0.79 points. F[1,37]=8.6 p=0.006  QoL at 6 months. QoL increased for men in the intervention group cf control. QoL scores for men receiving the intervention increased by 0.50 points whereas men in the control dropped by 0.84 points. F[1,37]=7.4 p=0.01 |
| **Berglund** 2003,2007 [32,33]  **1.**  RCT  Four arm  Stratified by metastasis at inclusion, curative treatment, no metastases, no curative treatment  211 men  Uppsala  Sweden  **2.**  **Mean age** 69 years, range 43-86 yrs.  **Ethnicity:** No information  **Marital status** 80% married  **Disease stage:** Metastasis at inclusion, 20%, Curative treatment 36%, No metastases, no curative treatment 44%  **Treatment:** WW 36%, RP 24%, RT 10%**,** HT 24%**,** Unknown 7%  **3.**  Newly diagnosed (within 6 months) prostate cancer patients  **4.**  None declared  **5.**  No  **6.**  **○ ○ 🞫○🞫** | **Intervention 1**  “Between Men” information program (I group )  **n=55**  **Delivered to:** Men  **Delivered by:** Urology nurse although a urologist held sessions 3 & 4  **Intensity:** 7 weekly 75 min sessions  **Individualised therapy?** No  **Procedure:** Group (from 3 to 10 participants). Emphasis on giving the participants full information available about treatment, side effects and how to deal with the side effects. Ample opportunity to discuss concerns with the nurse or urologist (when present).  **Components of therapy**   1. **Education** 2. **Discussion with HP** 3. **Delivered to group**   **Intervention 2**  Physical training (Ph group)  **n=53**  **Delivered to:** Men  **Delivered by:** physiotherapist  **Intensity:** 7 weekly 60 min sessions  **Individualised therapy?** No  **Procedure:** included light physical training, 2: relaxation, breathing exercises, exercises of the pelvic floor. A booster session was held 2 months after training exercises (to refresh the patients’ memory about the different exercises).  **Components of therapy**  1. exercise  **Intervention 3**  Information & physical training (I/Ph group )  **n=52**  Details as above    **Components of therapy**  1.Education  2.Discussion with HP  3.Delivered to group  4.Exercise  **Control:**  Standard care’ **n=51** | 1.  **Hospital anxiety & Depression scale**  (HADS) -14 item  European  **Organisation for Research and Treatment of Cancer Quality of life** (EORTC QLQ-)C30  **2.**  Baseline, 6 & 12 mth follow up (Berglund 2007)  **3.**  4 patients dropped out during the programme. 2 patients were dissatisfied with their group assignment  Other dropouts: Could not arrange transportation: 3 Ph, 4 I,4 IPh,  Overall only 158 completed & 33 failed to return questionnaire: 12 Ph, 7 I, 6 IPh, 8 C, | HADS  Depression was similar for each group  HADS  Anxiety was similar for each group  QoL EORTC  EORTC was similar for each group |
| **Giesler** 2005 [34]  **1.**  RCT  Two arm  n=99 men n=99 spouses  Indianapolis, USA  **2.**  **Mean age**: 63.8 yrs  **Ethnicity:** 90% Caucasian  8% African American, 2% other  **Disease status**  Diagnosis of stage T1a–T2c prostate carcinoma  **Treatment:** 63% surgery, 28% RT, 9% BT  **3.**  Scheduled to undergo or to have undergone surgery, RT, or BT, to have a spouse/relationship partner willing to participate , enrolled within 2 weeks after the conclusion of therapy; age≥ 18 years to speak fluent English.  **4.**  none declared  **5.**  No  **6.**  **○ ○🞫○✓** | Nurse led, menu driven computer program based intervention  **n=48**  **Delivered to:** Men & spouses  **Delivered by:** Nurse intervener  **Intensity**: monthly for 6mths, first 2 visits in person, others by phone  **Individualised therapy?** Yes  **Procedure**  1^st^ visit (within 6wks of active treatment finishing) focussed on assessing and managing bowel & urinary function using computer program. Video tape & “tool kit” provided.  2^nd^ visit (1mth later)  Focussed on problems related to sexual functioning, cancer worry, dyadic adjustment, depression & other cancer related issues with computer program. Further sessions used to discuss issues & concerns not addressed effectively previously & any new issues  **Components of therapy**   1. Psychological approach-psychoeducation 2. Discussion with HP 3. Delivered to couple 4. Psychological approach-psychoeducation 5. Discussion with HP 6. Delivered to couple   **Control:**  ‘Standard care’ **n=51** | **1.**  **Prostate specific QoL**  (PcQoL) 52-item  Center for **Epidemiologic Studies Depression Scale** (CES-D) 20-item  **Short form 36 for general QoL**  (SF-36) 8-item  **Dyadic adjustment**  (DAS)-32 item  **2.**  Baseline, 4, 7 & 12mths.  **3.**  By 12mth, 85 of the original dyads remained in study & 14 dyads had dropped out. Main reason given was inconvenience Attrition rates were similar across both groups. | **SF 36** There were no differences between the intervention or control group  **PCQoL**  There were no differences between the control group and intervention groups for urinary function, symptoms or bother.  **SF36 PCS**  Men in the intervention group had reduced role limitation due to sexual dysfunction compared to those in the control group at 7 and 4 months. Effect size = 0.50 p=0.02. Sexual bother and Sexual function were similar for both groups.  **PCQoL** Cancer worry  **CES-D**  Baseline depression was measured to see if it moderated effects of the intervention.  At follow-up there were no differences between the control or intervention group for depression (Effect size = 0.24 p=0.29) |
| **Lepore** 1999 [35]  **1.**  Pilot RCT  2-arm  24 men  Pittsburgh  USA  **2.**  **Age** No data given  **Ethnicity** 100% Caucasian  **Treatment** 83.3% RP, 20.9% RT, 12.5% HT  **Marital status** 91.7% married  **Cancer stage**  Stage A; 37.5%  Stage B: 50%  Stage C: 12.5%  **3.**  All men from 4 physicians’ offices who had had RP or RT for localised prostate cancer in April or May of 1996 were asked to participate.  **4.**  partly funded by Carnegie Mellon University development fund & NIH  **5.**  No  **6.**  **🞫🞫🞫🞫 ○** | Psychoeducational support group intervention  **n=12**  **Intervention was delivered to:**  Men & their wives. 7/11 wives of married men attended meetings  **Delivered by:** Lecture by expert of the week. There were two facilitators: a male clinical psychologist & female oncology nurse who shared responsibility for group dynamics.  **Intensity:** 6 weekly meetings of 2hrs each  **Individualised therapy:** No  **Procedure**  Prior to intervention men were interviewed in a face to face structured interview in their home (T1) and then 2 wks post intervention (T2) Men missing the lectures were sent audiotapes & copies of the hand outs  During discussion men (psychologist) and women (nurse) were separated.  **Components of therapy**  **1.**Education  **2.** Peer-discussion  **3.** Delivered to couple  **Control:**  **n=12**  Men did not attend a series of meetings but had the same T1 and T2 interview for outcome measures | **1.**  **General quality of life**  (SF-36 item)  **Self-efficacy**  (Investigators own measure)  **Social support**  (UCLA Social support Scale)  **2.**  T1= 1-2 weeks before intervention  T2 = 2 weeks post intervention  **3.**  2 men dropped out before T1. No attrition after T1. Most men attended most meetings Mean 5.7 meetings. | **SF36 PCS**  No differences between intervention groups  **SF36-MCS**  Men in the intervention group had greater gains in mental health scores over time (+14.33) compared with control. ( +0.67) (F(1,22)=5.15 p<0.05).  This was moderated but social support from family and friends.  The men who were dissatisfied with wife support or reported low support from family and friends had poorer MCS scores.  Self-efficacy coping  Study team’s own measure. The intervention group had greater increase in self-efficacy than the control group. |
| **Lepore** 2003 [36]  **Helgeson** 2006 [37]  **1.**  RCT  Three arm  279 men  Columbia  USA  **2.**  **Mean age:** 65yrs  **Ethnicity:** 90.4% Caucasian  9.2% African American 0.5% Asian American  **Marital status:** Married 87%  Separated/divorced/single 13%  **Treatment**: RP 57%, RT 17%, BT 17.5%, RT &BT 4.8%, Cryosurgery 4%  **Cancer stage:** T1 18%, T2 69%, T3 13%  **3.**  No history of other cancer. Living within 1hr drive of intervention site. Non metastatic disease at the time of study  **4.**  NIH Grant  **5.**  No  **6.**  **🞫🞫🞫○ ✓** | **Intervention 1**  Group education intervention (GE)  **n=84**  See detail below  **Intervention 2**  Group education intervention (GED) plus discussion  **n=86**  **Delivered to:** Men  **Delivered by:** For both GE & GED the lecture was delivered by an expert. FOR GED , discussion was led by a male clinical psychologist [ at this point female family members were mentioned – they had discussion in a different room] GED is GED plus 45 mins discussion  **Intensity:** GE six weekly 1hr sessions  **Individualised therapy?:** No  **Procedure:** Intervention 1 (GE) six weekly 1hr lectures & 10 minutes for questions. Intervention 2 (GED) GE plus 45 mins group discussion. Overview of prostate cancer biology and epidemiology (oncologist), control of physical side effects (urologist), nutrition and cancer (dietician), stress and coping (oncology nurse), relationships and sexuality (clinical psychologist), and follow-up care and future health concerns (urologist). Men also received printed materials summarizing the lectures.  **Components of therapy**  **1**. Education  **2.** Peer**-**discussion  **3.** Delivered to group  **Control:**  ‘standard medical care’ **n=80**  Plus baseline & follow up interviews | **1.**  **Health behaviour index**  (five questions by authors)  **General QOl**  (SF-36 )  **Disease specific QoL**  (UCLA -PC)  **Depression**  (Modified CES-D)  **Self-esteem**  Rosenberg self-esteem  **Self-efficacy**  Scale developed by authors  **2**.  Baseline, 2,wks, 6 &12 months post intervention  **3.**  29 (10%) were lost to follow-up. Reasons: major illness (4), death (2), time constraints (1), loss of interest (6), patient could not be located (2), and unexplained dropouts (14). | **SF36 PCS**  The SF36 PCS was similar for each of the groups although ANCOVA showed a ‘marginally significant effect of condition F(2,233)=2.35 p<0.10, and there was a significant group/education interaction where men with college degrees had significantly better PCS compared with those without a college education F(2,233)=2.98 p=0.05. There was no condition Educational status interaction F(2,233) =0.13 p=0.88  **UCLA OPC**  Urinary functioning was not affected by the intervention or interaction between the intervention with educational status or period  **UCLA OPC**  Sexual bother was reduced for men in the intervention group F(2,222) = 7.09 p<0.01 with those receiving education plus discussion mean=50.94 SE=2.99, compared with control mean=34.56 SE 3.13 and the men in the education only group had marginally less sexual bother than control p<0.10 although this was worse than the control and education plus discussion groups.  Sexual functioning was not affected by the intervention or interaction between the intervention with educational status or period  **Modified CES-D**  There were no differences between groups for depression F(2,235) =0.73 p=0.48. |
| **Manne** 2011 [38]  **1.**  Pilot multi-centre RCT  Two arm  71 men & partners from two cancer centres  New York  USA  **2.**  **Mean age:** 60yrs SD 8.3 for men 55.7 SD 8.5 for partners  **Ethnicity:** 89% white 11% non-white for men; 83% white & 11% non-white & 6% no description for partners  **Treatment:** 62% surgery ,28% RT, 0%R&HT,4% HT 6% not had treatment yet  **Marital status:** 97% married 3% co-habitating  **3.**  Localized prostate cancer diagnosed in the last year. ECOG performance status of 0 or 1. Married or living with a significant other of either gender.  Survivors & partners had to be ≥18yrs old. Live within a 2 hour commuting distance of the recruitment centre. English speaking. Not have a hearing impairment.  **4.**  Not declared  **5.**  No  **6.**  **○○🞫○✓** | **n=37**  Intimacy-Enhancing Therapy (IET)  **Delivered to:** Men & their partners  **Delivered by:** Five therapists provided the intervention. Therapists had 5 hours of training from the manual-based IET  **Intensity:** Five 90min couple sessions (dyads)- within 8 weeks  **Individualised therapy?:** Yes  **Procedure:** Five 90 minute couples’ sessions. The treatment was manualized Content focused on improving couples’ ability to comfortably share their thoughts & feelings regarding cancer, promote mutual understanding and support regarding their own & one another’s cancer experience, facilitate constructive discussion of cancer concerns, & to enhance and maintain emotional intimacy.  **Components of therapy**  **1.**Psychological approach- psycho-education [for couple intimacy]  **2.** Delivered to couple  **Control:**  ‘standard psychosocial care’  **n=34**  Same at both sites: Social work consultations are routinely provided for all survivors at both centres. If indicated, a referral to a psychiatrist or psychologist was provided by physicians at each site | **1.**  **Psychological distress**  (PD scale of the Mental Health inventory 24 items)  **Psychological well-being** (PWB scale of the Mental Health inventory -15 items)  **Cancer–specific distress**  (IES-15 items)  **Cancer concerns**  (authors own score)  **Relationship satisfaction**  (DAS 32 item)  **Relationship intimacy**  (PAIR 6 item scale)  **Erectile, bowel and urinary function**  (Erectile function domain of IIEF 6 items, urinary & bowel scales of UCLA PC index)  Measured at baseline & after sessions or at 8 weeks post baseline  **3.**  73% IET couples attended 4 or 5 sessions. ~5% of IET participants attended 1-3 sessions. 21.6% IET participants attended no sessions after being assigned to IET (pre-treatment drops). | **Psychological well-being**  **data**  **Cancer concerns** (authors own score)  There were no differences between groups  **Relationship satisfaction** (DAS 32 item)  There were no differences between groups  **Relationship intimacy** (PAIR 6 item scale)  There were no differences between groups  HADS  Depression was similar for each group  HADS  Anxiety was similar for each group |
| **Weber** 2004 [39]  1.  Pilot RCT  Two arm  30 men  Florida  USA  **2.**  **Mean age:** 58yrs  **Ethnicity:** 82% white, 13% white, 5% Hispanic  **Marital status** 90% married, 10% divorced/widowed/single  **Treatment**  Recent RP treatment  **3.**  Men who had recently undergone RP for prostate cancer (recruited 6wks after surgery). 45yrs or older. English speaking  **4.** Grants and awards from The Oncology Nursing Foundation, Rhone Poulenc Rorer Pharmaceuticals, The  Midwest Nursing Research Society, and the University Center on Aging and Health and the Alumni Association of the Frances Payne Bolton School of Nursing at Case Western Reserve University.  **5.**  No  **6.**  **○○🞫○○** | Trained peer buddy support  **n=15**  **Delivered to:** Men who had had a RP in the last 6wks  **Delivered by:**  Support partners 10 men (9 white & one black-mean age 68.2 years) who were long-term survivors of prostate cancer (>3yrs) & recruited to act as dyadic support partners. They were required to have stable PSA levels, be English speaking and had a RP that resulted in urinary and sexual dysfunction. They participated in a 2-h training where skills & possible topics of dyadic discussion were talked about.  **Intensity:** These dyads meet 8 times in 8 wks  **Individualised therapy?:** Yes  **Procedure**  The meetings took place at gourmet coffee shops with the relaxed atmosphere necessary for two men  to have a private conversation without feeling rushed or intimidated.  Support partners recorded duration of meetings, the focus of discussions, & the quality of meetings in weekly logs.  **Components of therapy**  1.Trained peer buddy system  2. Delivered to Individual  **‘**Usual care’  **n=15** | **1.**  **Social Support**  (Modified Inventory of Socially Supportive Behaviours 41 items)  **Self–efficacy**  (SIC-PA, 38 item)  Both of the above at baseline & 8wks  **Depression**  (GDS- 15items)  Baseline, 4 & 8 wks  **Incontinence & erectile dysfunction**  (UCLA Prostate cancer Index)  **2.**  Baseline & 8wks  **3.**Two men dropped  Out from intervention group before study started  All 15 men randomly assigned to the dyadic support program completed the 8 weeks | **SIC-PA**  Baseline imbalance with control having greater self-efficacy mean control 319.5 intervention 290.3 t=2.5, p=0.05  However improvement was seen only in the Intervention group (Paired t-test, t=-2.2, p=0.04)  **UCLA**  No differences between groups for urinary function, urinary bother  **UCLA**  No differences between groups for sexual function, but at 8 weeks dyadic support group had less sexual function bother (t=2.6, p=0.014)  **GDS**  Level of depressive symptoms was similar for both groups at 8 weeks (F=2.22, p=0.14) but at 4 weeks the intervention group had fewer depression symptoms (F=5.90 p=0.02) [Both adjusted for baseline depression and self-efficacy]. |
| **Weber** 2007 [40]  **1.**  RCT  Two arm  69 Men  Florida  USA  **2.**  **Mean age** 60yrs SD 7.0  **Ethnicity:** 82% white, 11% black, 4% Hispanic & 3% Asian  **Treatment:** Men have had RP in the last wks  **Marital status:** 74% married,  26% Single/widowed/divorced  **3.**  Diagnosed with prostate cancer, Aged 45yrs plus,  Recently treated with RP (within 6wks), Within 3mths of cancer diagnosis, No psychiatric disorders,  English speaking  **4.**  National Cancer Institute  **5.**  No  **6.**  **○○🞫○○** | Trained peer buddy support  **n=37 (n=35 followed up)**  **Delivered to:** Men who have had a recent RP  **Delivered by:** 11 men (9 white men & 2 black men) who underwent RP ≥3 years prior were identified & trained to provide dyadic support from a prostate cancer survivor’s point of view. Support partners were identified by participating urologists from a pool of  former patients who were thought to have successful recovery from prostate cancer treatment & who experienced similar side effects as the research participants. The support partners were trained to recognize signs & symptoms of  clinical depression as defined by the *DSM-IV*, communicate with active listening  skills.  **Intensity:** The dyads met 8x during an 8-weeks  **Individualised therapy?:** Yes  **Procedure:**  In addition to usual health care, the men were paired (dyad) with a former patient (support partner) The dyads met and the support partner was able to vicariously relate successful coping & recovery strategies after prostatectomy. The meetings were held at gourmet-style coffee shops where the men were able to discuss issues & concerns in a semi-private environment that had living room style seating. This setting provided an opportunity for the men to talk in a relaxed atmosphere without feeling rushed.  **Components of therapy**   1. Trained peer buddy system 2. Delivered to individual   **Control:** ‘Usual health care’  **n=32** | **1.**  **General health related QoL**  (SF-36 ).  **Prostate cancer specific QoL**  (UCLA prostate cancer Index 20-item)  **Self-efficacy**  (Stanford Inventory of Cancer Patient Adjustment 38-item)  **Depression**  (GDS-15 item)  **2.**  Baseline & 8 weeks  **3.**  7 lost to follow-up before any data collection (2 experimental & 5 controls) & 2 patients relocated Thus, attrition was 8.6% & 72 men. Completed survey data at post-test. | UCLA-prostate cancer index  Control (Mean=49.3 SD 28.8) and intervention (Mean 29.5 SD 18.9) had different urinary function at baseline. Mann Whitney U =370 p=0.04. Urinary bother were similar at a baseline. No report of the difference between these groups at follow up  UCLA-prostate cancer index. Sexual function and sexual bother were similar at baseline. No report of the difference between these groups at follow up.  Self-Efficacy  Men in the intervention group (mean = 328.89 SD = 40.60) had greater scores for self-efficacy compared with the control (mean 300 SD 43.76) At 8 weeks p=0.005  No men had clinically depression warranting referral to further care. Men in the intervention group (mean 0.92 SD 1.32) had reduced depression compared with the control (mean = 2.53 SD = 3.662) at 8wks p=0.005. This effect persisted when baseline self efficacy and depression were controlled for F=4.854 p=0.32) and when baseline urinary and sexual function and bother were controlled for (F=4.9 p=0.031).  Data from SF36 were skewed and could not be transformed and were not analysed |

**Table 2c:** Supportive care interventions for men with prostate cancer longer term post primary treatment

| **Study Details and Participants**  1.Study type  2.Baseline characteristics  3.Eligibility criteria  4.Funding  5. Power calculation  6.Risk of bias* 5 domains | **Intervention and Control group(s)** | **Data collection** 1.Relevant outcome measures  2.Follow-up  3.Attrition | **Results** |
| --- | --- | --- | --- |
| **Campbell** 2007 [41]  **1.**  Pilot RCT  Two arm. 40 couples  North Carolina, USA  **2.**  **Mean age:** 62 yrs  **Ethnicity:**100% African American  **Treatment:** RP 93%, HT(inject) 12.5%, HT (pill) 7%, WW 7%, RT 17%(control grp only)  **3.**  African-American men beyond acute diagnosis  & treatment phase for prostate cancer. KPS score 44 -60 (relatively independent).With an identified intimate partners (spouse or individual or committed relationship.  **4.**  National Cancer Institute  5.  No  **6.**  **🞫🞫🞫🞫🞫** | **n=20**  Telephone-based coping skills training  **Delivered to:** Men & their partners  **Delivered by:** African American, doctoral level medical skilled in CST, trained on the 6-session protocol. **Intensity:** 6 weekly, 1 hour phone sessions. **Individualised therapy?:** Yes  **Procedure**  *Session 1:* Rationale for CST given. Couples identified specific challenges & describe existing coping style. Coping skills were taught and modified for each couple. Therapists assessed couple’s need for basic education about prostate cancer & began CST by introducing problem solving.  *Session 2:* Progressive muscle relaxation (PMR) training.  *Session 3:* Communication skills *Session 4:* Activity/rest  cycling (to pace activities to manage fatigue, pain, or other symptoms) using goals aim to increase pleasant activities.  *Session 5*: cognitive restructuring to identify irrational, maladaptive thought & to replace these with rational coping thoughts. Imagery taught as enhancement to PMR.  Session 6: A coping maintenance plan developed with each couple. The interventionist briefly interviewed each couple about their experience with CST. Delivered over the phone with loud speaker provided for ease.  Manualised: Yes  Theory based: Yes  **Components of therapy**  1.Education  2.Psychological approach: cognitive restructuring  3. Relaxation therapy  4. Intervention delivered to couple  **Control:** ‘Routine care for couple’  **n=20** | **1.**  **Self-efficacy**  (SESCI– 3 subscales)  **Prostate cancer specific QoL**  (EPIC 50 item)  **General health QoL** (SF-36)  **2.**  Pre and post treatment over telephone  **3.**  15/20 couple completed intervention of the 15, two provided pre-treatment data only, one couple‘s data was considered outlying & was excluded. Control grp , 2 couple provide pre-treatment data only | **1.**  **Self-efficacy**  (SESCI– 3 subscales)  No difference between intervention and control.  **Prostate cancer specific QoL**  (EPIC 50 item)  Urinary Function: EPIC 50 item  No difference between intervention and control  Erectile function:  EPIC 50 item  No difference between intervention and control  Hormonal bother:  EPIC 50 item  No difference between intervention and control  **General health QoL** (SF-36)  No difference between intervention and control  PCS Int Mean = 27.52 (SE = 0.814)  Con Mean = 26.10 (SE =0.664) p 0.192 (cohen’s d = 0.341)  MCS Int Mean = 24.90 (SE = 0.664)  Con Mean = 25.24 (SE = 0.54) p 0.699 (cohen’s d = 0.008) |
| **Molton** 2008 [42]  **1.**  RCT 2-arm, Florida USA, 121 men  **2.**  **Age:** 60.3 years SD 4.9;  **Ethnicity:** White 43%, Hispanic 38% African Am 18%.  **Treatment:** RP=100%  **Marital status:** 78%  **3.**  At least 45 years old. RP for Stage I or II prostate cancer and 9^th^ grade reading level free of cognitive impairment or active psychiatric symptoms including panic attacks PTSD or alcohol or drug dependence in last 3 months. No prior history of cancer no adjuvant treatment for prostate cancer. Received treatment more than 18 months ago.  **4.**  National Cancer Institute  **5.**No  **6.**  **○ ○🞫○🞫** | Cognitive Behavioural Stress Management (CBSM )  **n=60**  **Delivered to:** Men in groups of 4-6 participants.  **Delivered by:** 2 therapists or practitioners. Masters level clinical health psychology students and or parallel PhD level licensed clinical psychologists trained in the CBSM protocol.  **Intensity:** 10 weeks /1 per week/ 2 hours.  **Individualised therapy?:** No  **Procedure:**  90 minute CBSM didactic instruction plus discussion plus 30 minute relaxation. Intervention was designed to provide participants with skills to manage day-to-day stressors as well as manage PC-associated physical & emotional challenges, including sexual dysfunction,spousal or partner relations, engagement of the medical system, by providing individuals with effective coping & stress-management techniques and practical information regarding prostate cancer treatment and recovery. Participants were encouraged to talk to one another about their experiences. Weekly homework and relaxation exercises were assigned to elaborate on session material.  The relaxation component varied and included training in deep-breathing,  guided imagery, progressive muscle relaxation, transcendental and mindfulness meditation.  **Manualised** : Yes (unpublished)  **Theory based:** not clear.  **Components of therapy**  1.Psychological approach: CBT for stress management & psycho-education  3.Relaxation therapy  3.Peer discussion  4. intervention delivered to group  5.Homework  **Control:** **n=41**  Met in groups for single 4 hour seminar. Provided with basic educational materials on stress management techniques. Details as for main intervention | **1.**  **Sexual functioning** UCLA-PCI  (plus Interpersonal sensitivity IIP-PD Inventory of Interpersonal Problems  & Personality Disorder (IIP-PD) scale were used in analysis as moderators)  **2.**2-3 weeks after intervention ended.  **3.** 121 men assessed at baseline. 101 completed all measures and are used in analysis. There is no information about the people who dropped out. | **Sexual functioning** UCLA-PCI  After controlling for baseline sexual functioning, age, medical co-morbidities, and use of sexual aids CBSM group assignment was a *good predictor of* post-intervention sexual functioning: Beta = 0.14 p<0.05 See Table 1 and fig1. .  Authors report: ‘Those in the experimental condition made a 37.4% improvement in sexual functioning compared with the control where improvement was only 11.5%.’  Post-hoc regression analyses were used to see if ‘Interpersonal sensitivity’ mediated change in sexual functioning. – it did – ‘those with higher levels of interpersonal sensitivity were particularly responsive to CBSM. ‘ |
| **Penedo** 2004 [43]  **1.** RCT, 2-arm, 92 Men  Florida USA  **2.**  **Age:** 63.1 yrs SD 2.2  **Ethnicity:** White 35%, Hispanic 34% African Am 22% other 9%.  **Treatment:** RP=65 RT =27  **Marital status:** Not stated  **3.**  At least 50 years old. Either RP or RT for Stage I or II (TNM TiA to t2b) prostate cancer in the last 18 months. 9^th^ grade reading level free of cognitive impairment or active psychiatric symptoms including panic attacks PTSD or alcohol or drug dependence in last 3 months. No prior history of cancer no adjuvant treatment for prostate cancer.  **4.**  National Cancer Institute  **5.**  No  **6.**  **○ ○🞫○○** | CBSM  **n=52**  **Delivered to:** as above **Delivered by:** as above  **Intensity:** as above  **Individualised therapy?** As above **Procedure:** As above but no mention of peer discussion  **Manualised:** Yes (Unpublished)  **Theory based:** Not clear  **Components of therapy**  1.Psychological approach –CBT for stress management & psycho-education  2.relaxation therapy  3. Intervention delivered to group  **Control: n=40**  Met in groups for a single 4 hour seminar on same stress management skills shown that were included in intervention. | **1. QoL** FACT-G | 1.  FACT G  Authors do not report a difference between the groups for QoL  Hierarchical regression modelling : Controlled for ethnicity, baseline QoL and income: Group assignment was *a significant predictor* of post intervention QoL (Beta=-0.14 p=0.03).  QoL was significantly increased for men who received the intervention from baseline mean 87.31 (SD 12.41) to post intervention mean 90.48 (SD=11.27) t=2.90 p<0.01).. In control group there was no difference between baseline (mean 89.37 SD 14.30) to follow-up (mean 88.65 SD 13.19) t=0.47 p>0.10.  Intervention was a successful predictor.  NB this is not a comparison of the 2 interventions – it is a comparing pre-treatment values at baseline to post treatment values..  Focus of paper on whether ethnicity can affect QOL but there was no evidence for this. |
| **Penedo** 2006 [44]  **1.**  RCT  2-arm  233 men (191 at follow-up)  Florida  USA  **2.**  **Age:** Int 64.9yr SD 7.64; Control 65.30 SD 7.8.  **Ethnicity:** White 39%, Hispanic 39% African Am 11% other 6.5%.  **Treatment:** RP=65 RT =27  **Marital status:** 74% married, 16% separated /divorced/widowed  **3.**  At least 45 years old. Recruited years 2000 to 2004. Either RP or RT (including EBRT and brachytherapy) for Stage I or II prostate cancer in the last 18 months. 9^th^ grade reading level free of cognitive impairment or active psychiatric symptoms including panic attacks PTSD or alcohol or drug dependence in last 3 months. No prior history of cancer no adjuvant treatment for prostate cancer.  **4.**  National Cancer institute  **5.**  No  **6.**  **○ ○🞫○🞫** | CBSM  **n=133**  **Delivered to:** as above  **Delivered by:** as above  **Intensity:** as above  **Individualised therapy?:** as above  **Procedure**  As above  **Components of therapy**  1.Psychological approach –CBT for stress management & psycho-education  2.relaxation therapy  3.Intervention delivered to group  4.Peer discussion  **Control: n=100**  Met in groups for a single 4 hour seminars on same stress management skills shown that were included in intervention. | **1.**  **Quality of Life**  FACT-G  **Benefit Finding**  **Positive Contributions Scale Cancer**  (PCS-C)  **Perceived stress management skills** PSMS  **2.**  2-3 weeks after the intervention ended  **3.**  134 men were allocated to the CBSM intervention. 1 became ineligible before treatment. 8 missed follow-up, 11 dropped out & 7 became ineligible  101 men were allocated to the control. 1 withdrew before treatment started. 5 missed follow-up, 6 dropped out and 5 became ineligible. | 1. QoL  Post hoc analyses. Participants in the experimental condition experienced significant pre-intervention to post intervention increases in QoL (t=2.65, P<0.01).  Assignment to CBSM or control was a significant  predictor of post-intervention QoL (F=4.32 p<0.05) and PSMS (F=11.02 p<0.01) and BF (F=5.52 p<0.05).  Post hoc analyses showed CBSM had significantly better pre-post intervention increases in QoL (t=2.65, p<0.01); BF (t=2.65, p<0.01) and PSMS (t=3.35, p<0.01).  2.  Self-efficacy of cognitive restructuring Benefit finding Positive contributions scale (PCS)  Post-hoc analysis revealed that participants in the experimental condition experienced significant pre-intervention to post intervention increases in Benefit Finding (t=2.65, P<0.01) and Perceived Stress Management Skills (PSMS) t=3.35, p<0.01 |
| **Traeger** 2013 [45]  **1.**  RCT  2-arm  257 Men  Florida USA  **2.**  **Age:** 65.3 years SD 7.7 years  **Ethnicity:** White 40.9%, Hispanic 42% African Am 16.7%.  **Treatment:** RP=47.1% RT =52.9%  **Marital status:** 75.1% in a relationship.  **3.**  At least 50 years old. Either RP or RT for prostate cancer. 9^th^ grade reading level free of cognitive impairment or active psychiatric symptoms including panic attacks PTSD or alcohol or drug dependence in last 3 months. No prior history of cancer no adjuvant treatment for prostate cancer.  **4.**  Not stated  **5.**  No  **6.**  **○ ○🞫○○** | CBSM  **n=148**  **Delivered to:** as above  **Delivered by:** as above  **Intensity:** as above  **Individualised therapy?:** as above  **Procedure**  As above  **Components of therapy**  1.Psychological approach –CBT for stress management & psycho-education  2.relaxation therapy  3.Intervention delivered to group  4.Peer discussion  **Control:**  **n=109**  Met in groups for a single 4 hour seminars on same stress management skills shown that were included in intervention. | **1.**  **QoL**  FACT-G (27-item)  **Illness perception Questionnaire**  IPQ-R (items not stated)  **Sexual and urinary dysfunction**  EPIC (items not stated)  **Perceived Stress Scale**  PSS(14 item)  **2.**  2-3 weeks after the intervention ended  **3.**  Of n=148 CBSM 5 were lost to follow-up & 20 dropped out. 123 were analysed.  Of 109 controls, 8 lost to follow up and 9 dropped out. 92 were analysed. | 1. QoL  FACT-G  Only data from one scale presented: Emotional well being scale.  Participants who received CBSM had better QoL than those in control group.  Standardised coefficients = 0.13 p<0.05 and controlling for pre EWB – standardized coefficient = 0.52 -<0.01 and SES coefficient 0.009, p>0.005.  EPIC measured but not reported.  Authors state  “As hypothesized, CBSM participation led to greater  post-intervention emotional well-being relative to participation  in a half-day psycho-educational seminar.  This effect was independent of sexual and urinary dysfunction  at study entry. **“**  But caution in interpreting these due to risk of bias score for this study. |

**Table 2d:** Studies involving men at all stages of prostate cancer & treatment (n=1)

| **Author**  1.Study type  2.Baseline characteristics  3.Eligibility criteria  4.Funding  5. Power calculation  6. Risk of bias* ○=Unclear risk ✓=Low risk 🞫=High risk | **Intervention and Control group(s)** | **Data collection**  1.Relevant outcome measures  2.Follow-up  3.Attrition | **Authors’ results** |
| --- | --- | --- | --- |
| **Northouse** 2007 [46]  **1.**  RCT  2- arm  n=263 patient-spouse dyads  Stratified by treatment centre & phase of illness  Michigan USA  **2.**  **Mean age:** men 63yrs (49-90yrs) spouses 59yrs (34-84yrs)  **Ethnicity:** 84% of dyads were Caucasian , 14% African American ,2% others  **Cancer stage/treatment**  65% newly diagnosed of which 60% had RP & 40% EB-RT  14% were in biochemical recurrence with 50% on observation 50% treatment (mainly hormone)  21% were advanced cancer with 36% HT& 64% refractory hormone treatment  **3.**  Newly diagnosed after completion of primary treatment or biochemical recurrence after 2 consecutive rises in their PSA or advanced prostate cancer after diagnosis or progression of metastatic disease. Age ≥30yrs. A life expectancy of >12mth.  A spouse or live in partner. Living within 75 miles of participating centres. Patients with a second primary cancer were excluded. **Spouses or Partners**: Age ≥21yrs & identified by PHC provider as emotional & physical supporter of men. Couples were excluded if spouses had been diagnosed with cancer within the prior year and were receiving treatment  **4.**  NCI with additional funds from Fashion Footwear Charitable Foundation of NY/ QVC Presents Shoes on Sale™ and University of Michigan Comprehensive Cancer Center, Office of Research & Sponsored  Programs & School of Nursing.  **5.**  Yes  **6.**  **○ ○🞫✓○** | FOCUS program, a supportive-educative intervention  **n=129**  **Delivered to:** Men– spouses dyads  **Delivered by:** Masters trained N=nurses specifically trained by PI & co-investigators during 40 hour training program , viewed a FOCUS intervention training video & accompanied nurses on home visits prior to having their own workloads  **Intensity:** Three 90 min home visits & 2 30 min telephone sessions 2 wks apart within a maximum of 4mths. **Individualised therapy?:** Yes  **Procedure:** FOCUS intervention comprised Family involvement, **O**ptimistic attitude, **C**oping effectiveness, **U**ncertainty reduction & **S**ymptom management. First developed for breast cancer patients & spouses and modified for prostate cancer patients& spouses **Manualised:** Yes  Theory based: not described  **Components of therapy**  1.psychological approach-psycho-education [self-care and coping]  2. Intervention delivery to couple  **Control:** ‘Standard clinic care’ n=134  Some centres offered support groups but there were no specific psychosocial resources targeted for couples facing prostate cancer | **1.**  **General QoL**  (MOS-SF-12 version 2)  **Cancer specific QoL** (FACT-G version 4 27-item  **Prostate specific QoL**  (FACT-P)  **Coping strategies** (Brief coping orientations to problems experienced Scale 28 item)  **Self-efficacy**  (Lewis Cancer Self-efficacy Scale 17 item)  **General symptom distress** (OSQ)  **Prostate specific symptoms**  (EPIC -50 item)  2.  Baseline, 4, 8 & 12 months follow up  **3.**  90% completed 4mths 83% completed all 3 follow up assessments | There were no significant effects of the treatment group vs. control  [ there were sustained effects on Qol for spouses up to 12 mths]  *Authors state*  “men with prostate cancer and their spouses reported positive outcomes from the intervention . Programs of care need to be extended to spouses who likely will experience multiple benefits from intervention.” |

**Foot notes:**

*****Risk of bias: 5 domains are scored as ○=Unclear risk ✓=Low risk 🞫=High risk. In order the domains measured are *Selection bias:* 1. Random sequence allocation, 2. Allocation concealment, *Performance bias* 3. Blinding of participants and personnel, *Detection bias:* 4. Blinding of outcome assessments, *Attrition bias:* 5. Incomplete outcome data

**Abbreviations:**

ADT Androgen deprivation therapy

BF Benefit finding (post-traumatic growth, cognitive restructuring)

BPI Brief Pain Inventory

CBT Cognitive Behavioural Therapy

CBSM Cognitive Behavioural Stress Management

CES-D Center for epidemiologic studies – depression scale

CR Cognitive reframing

CST Partner assisted coping skills training

DAS Dyadic adjustment scale

EBRx External beam radiotherapy

EPIC Expanded prostate cancer index composite

EORTC QLQ-C30 European Organisation for Research and Treatment of Cancer Quality of life

FACT-G Functional Assessment of Cancer Therapy – General

Fact-P Functional Assessment of Cancer Therapy – For people with prostate cancer

GDS Geriatric Depression Scale

GE Group education intervention

GED Group education intervention plus discussion

GTUS Growth through uncertainty Scale 39 items – 4 domains

HADs Hospital anxiety & Depression Scale 14 item

HT Hormone therapy

HP Health professional

HMT Hormone maintenance therapy

IIED (International index of Erectile dysfunction

IIP-PD Inventory of Interpersonal Problems & Personality Disorde

I “Between Men” information group

I/Ph “Between Men” Information & physical training (I/Ph group )

IPQ-R Illness Perception Questionnaire (revised)

JCS-40 Jalowiec coping scale (40 item)

KPS Karnofsky performance Status scores

MOS-SF-12 The Medical Outcomes Study: Measures of Quality of Life Core Survey – Short form 12

mths Months

PC Prostate Cancer

PCS-C Positive Contributions Scale Cancer

PCQoL Prostate cancer Quality of Life

Ph Physical training group

PMR Progressive muscle relaxation

POMS Profile of mood states

Profile of Mood States – Short Form (POMS) 37 items 6

POMS BI 72 Profile of mood states Bi-polar (72 item)

PSMS=Perceived stress management skills

PSS=Perceived stress scale

PTSD Post traumatic stress disorder

QoL=Quality of Life

RCT Randomised controlled trial

RP radical prostatectomy

RT Radiotherapy

RRT Relaxation response therapy

SC Standard care

SD Standard deviation

SESCI Self efficacy for symptom control

SICA-PA Stanford Inventory for Cancer Patient Adjustment

SM pre-surgical stress management intervention

SA pre-surgical “supportive attention” intervention

SF-36 PCS the Short Form (36) Health Survey – Physical component scale

SF-36 MCS the Short Form (36) Health Survey – Mental component scale

STAI State-Trait Anxiety Inventory

TURP Trans urinary resection of the prostate

UCLA-PCI University of California Los Angeles – Prostate Cancer Index
